# Carcinogen exposure enhances cancer immunogenicity by blocking the development of an immunosuppressive tumor microenvironment

**DOI:** 10.1172/JCI166494

**Published:** 2023-10-16

**Authors:** Mei Huang, Yun Xia, Kaiwen Li, Feng Shao, Zhaoyi Feng, Tiancheng Li, Marjan Azin, Shadmehr Demehri

**Affiliations:** 1 Center for Cancer Immunology and Cutaneous Biology Research Center, Department of Dermatology and Center for Cancer Research, Massachusetts General Hospital and Harvard Medical School, Boston, Massachusetts, USA.; 2 Department of General Surgery, The First Affiliated Hospital of USTC, Division of Life Sciences and Medicine, University of Science and Technology of China, Hefei, Anhui, China.

**Keywords:** Oncology, Cancer immunotherapy, Innate immunity, Macrophages

## Abstract

Carcinogen exposure is strongly associated with enhanced cancer immunogenicity. Increased tumor mutational burden and resulting neoantigen generation have been proposed to link carcinogen exposure and cancer immunogenicity. However, the neoantigen-independent immunological impact of carcinogen exposure on cancer is unknown. Here, we demonstrate that chemical carcinogen-exposed cancer cells fail to establish an immunosuppressive tumor microenvironment (TME), resulting in their T cell–mediated rejection in vivo. A chemical carcinogen-treated breast cancer cell clone that lacked any additional coding region mutations (i.e., neoantigen) was rejected in mice in a T cell–dependent manner. Strikingly, the coinjection of carcinogen- and control-treated cancer cells prevented this rejection, suggesting that the loss of immunosuppressive TME was the dominant cause of rejection. Reduced M-CSF expression by carcinogen-treated cancer cells significantly suppressed tumor-associated macrophages (TAMs) and resulted in the loss of an immunosuppressive TME. Single-cell analysis of human lung cancers revealed a significant reduction in the immunosuppressive TAMs in former smokers compared with individuals who had never smoked. These findings demonstrate that carcinogen exposure impairs the development of an immunosuppressive TME and indicate a novel link between carcinogens and cancer immunogenicity.

## Graphical abstract

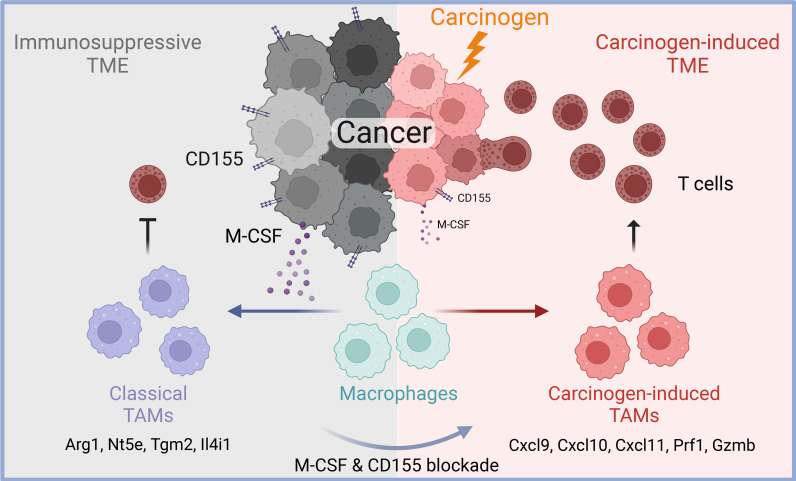

## Introduction

The advent of immune checkpoint blockade (ICB) therapy has greatly improved cancer treatment, especially for metastatic melanoma, non-small cell lung cancer and squamous cell carcinoma of the head and neck (1–3). However, many cancer types like breast cancer do not respond to ICB therapy (4). Across 27 cancer types, the objective response rate to PD-1/PD-L1 blockade therapy is positively correlated with tumor mutational burden (TMB) (5). Tumors with high TMB show greater cytotoxic immunity in the tumor parenchyma, denoting them as immunologically “hot” tumors (6). Importantly, cancers with high TMB are caused by exposure to environmental carcinogens (7). Thus, it is critical to determine the mediators of increased cancer immunogenicity by carcinogens and examine their therapeutic utility to improve the response of “cold” tumors to immunotherapy.

The prevailing mechanism linking carcinogen exposure to enhanced cancer immunogenicity posits that high TMB caused by the carcinogen results in an increased neoantigen load thereby generating “hot” tumors responsive to ICB therapy (8). Neoantigens are nonself antigens created by nonsynonymous somatic mutations in tumor cells, which are found to boost tumor-specific T cell response and increase cancer immunogenicity (9). This paradigm provides an explanation for why UV-induced cutaneous melanoma is highly responsive to ICB therapy (10). The response of more than half of all cancers (55%) to ICB therapy can be explained by TMB and putative neoantigen load (5, 11). In addition, the induction of TMB and associated neoantigens on cancer cells by carcinogen exposure has been shown to elicit functional T cell response and increase cancer immunogenicity in both in vivo and in vitro models. For instance, melanoma cell line treated with UV radiation (YUMMER1.7) exhibits higher immunogenicity associated with increased TMB compared with the parental YUMM cell line (12). However, only a subset of cancers with high TMB and neoantigen load show a durable response to immunotherapy (13). In addition, increasing TMB/neoantigen to improve cancer immunogenicity is not a viable therapeutic strategy due to the detrimental cell-autonomous impacts of increased TMB on cancer progression and metastasis. Thus, it is essential to determine the full immunological impact of carcinogen exposure on cancer cells to uncover potential pathways that are suitable for therapeutic intervention.

Herein, we investigated the immunological impact of chemical carcinogens on mouse breast and lung cancer cell lines, which consistently enhanced their immunogenicity. Strikingly, the most immunogenic carcinogen-treated cell line did not show any gain of somatic mutations. Careful examination of this carcinogen-treated cell clone and its vehicle-treated counterpart revealed that enhanced immunogenicity was not driven by the gain of an immunogenic factor. Instead, it was mediated by the loss of the immunosuppressive tumor microenvironment (TME), due, in part, to markedly reduced M-CSF expression by carcinogen-treated cancer cells. Accordingly, tumor-associated macrophages (TAMs) in tumors generated from carcinogen-treated cancer cells exhibited antitumor properties, while TAMs in the tumors generated from vehicle-treated cancer cells had a classical immunosuppressive phenotype. Importantly, the carcinogen-induced TAM population dominated the lung cancers of smokers, who are known to be more responsive to ICB therapy (14). Our findings reveal a neoantigen-independent impact of carcinogens on cancer immunogenicity through the modulation of TAMs, which we believe can be therapeutically utilized to improve cancer immunotherapy.

## Results

### Carcinogen exposure increases cancer cell immunogenicity.

**Figure 1:**
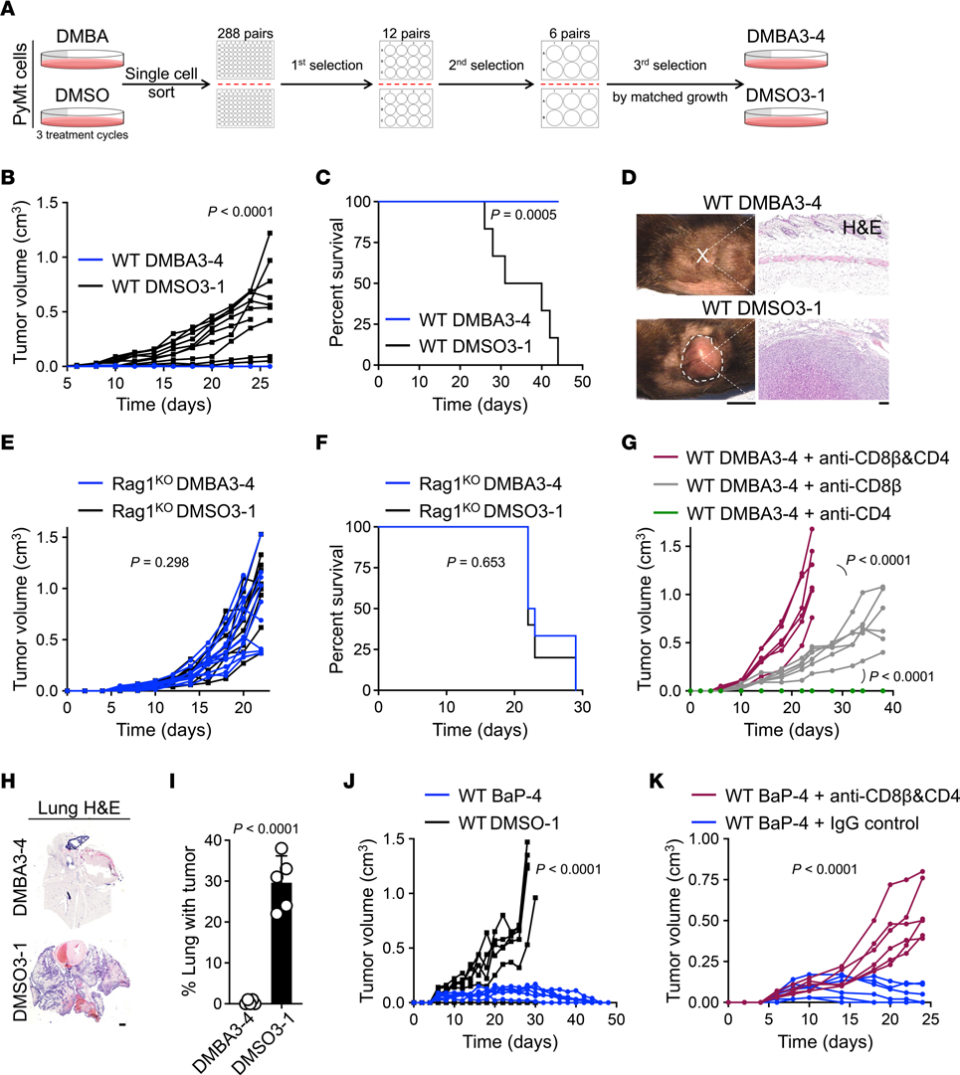
PyMt cells acquire high immunogenicity upon carcinogen exposure. (**A**) Schematic diagram of DMBA3-4 and DMSO3-1 cell clones derived from DMBA and DMSO (vehicle control) exposed PyMt cell line, respectively. (**B** and **C**) DMBA3-4 and DMSO3-1 tumor kinetics in syngeneic WT C57BL/6 mice shown as (**B**) tumor growth over time (*n* = 10 per group) and (**C**) animal survival rate (*n* = 6 per group). (**D**) Representative macroscopic and H&E-stained histological images of DMBA3-4 and DMSO3-1 tumor injection sites in WT mice when DMSO3-1 tumors reach terminal size. Note the absence of any DMBA3-4 tumor in the s.c. fat pad. Scale bars: 1 cm, mouse; 100 μm, histology. (**E** and **F**) DMBA3-4 and DMSO3-1 tumor kinetics in syngeneic Rag1^KO^ mice shown as (**E**) tumor growth over time (*n* = 12 for DMBA3-4 and *n* = 10 for DMSO3-1 group) and (**F**) mouse survival rate (*n* = 6 for DMBA3-4 and *n* = 5 for DMSO3-1 group). (**G**) DMBA3-4 tumor growth in WT mice treated with anti-CD8β, anti-CD4 depleting antibody alone or the combination of anti-CD8β and anti-CD4 antibodies (*n* = 6 per group). (**H** and **I**) Lung metastasis of DMBA3-4 and DMSO3-1 cells shown as (**H**) representative H&E-stained histological images of the lung (scale bar: 1 mm) and (**I**) percent lung surface area occupied by tumor foci at day 21 after i.v. injection of 200,000 cells per mouse (*n* = 5 per group). (**J**) BaP-4 and DMSO-1 tumor growth in syngeneic WT C57BL/6 mice (*n* = 6 per group). (**K**) BaP-4 tumor growth in WT mice treated with anti-CD8β and anti-CD4 combination antibodies versus IgG control antibody (*n* = 6 per group). Mice received 100,000 cancer cells per orthotopic injection site. 2-way ANOVA (**B**, **E**, **G**, **J**, and **K**), unpaired *t* test (**I**) and log-rank test (**C** and **F**), bar graph shows mean + SD.

To investigate the impact of carcinogen exposure on cancer cell immunogenicity, we treated MMTV-PyMt^tg^ (PyMt) breast cancer cell line (15) with 7,12-Dimethylbenz[a]anthracene (DMBA) carcinogen or DMSO as a vehicle control. After 3 treatment cycles, single cells were sorted and expanding clones were selected (Figure 1A). 6 pairs of DMBA- and DMSO-treated clones were compared for their proliferation rate in vitro (Supplemental Figure 1A; supplemental material available online with this article; https://doi.org/10.1172/JCI166494DS1). Among them, PyMt-DMBA3 clone 4 (DMBA3-4) and PyMt-DMSO clone 1 (DMSO3-1) showed a similar proliferation rate in vitro and were selected for follow-up in vivo studies (Figure 1A and Supplemental Figure 1B). We injected 100,000 DMBA3-4 and DMSO3-1 cells s.c. into the inguinal mammary fat pad of syngeneic WT C57BL/6 mice and monitored tumor growth over time. DMBA3-4 cells did not form any tumors in immunocompetent mice while DMSO3-1 cells generated tumors that reached terminal size by 44 days after injection (Figure 1, B–D). Injection of 250,000 and 500,000 DMBA3-4 cells also did not result in any tumor formation in immunocompetent mice (Supplemental Figure 1C). Importantly, 100,000 DMBA3-4 cells injected into T and B cell-deficient *Rag1^–/–^* (Rag1^KO^) mice formed tumors that grew at the same rate as DMSO3-1 cells (Figure 1, E and F). In addition, DMBA3-4 cells formed tumors in WT mice upon CD8^+^ T cell depletion but not CD4^+^ T cell depletion alone (Figure 1G). DMBA3-4 tumor growth was further accelerated in WT mice that underwent CD8^+^ T plus CD4^+^ T cell depletion (Figure 1G). In a metastasis model, i.v. injection of DMBA3-4 cells did not result in any tumor foci in the lung, while DMSO3-1 cells formed multiple tumor foci in the lung of immunocompetent WT mice (Figure 1, H and I). Collectively, these findings demonstrate that DMBA3-4 cells are rejected by T cells in vivo. Thus, history of DMBA exposure highly increases breast cancer cell immunogenicity.

Next, we examined whether a similar effect can be observed in other breast cancer cell lines and in exposure to other chemical carcinogens. We treated the 4T1 breast cancer cell line with DMBA versus DMSO and generated single cell clones (Supplemental Figure 1D). 4T1-DMBA6-3 cells showed limited tumor growth in syngeneic immunocompetent WT BALB/c mice compared with 4T1-DMSO6-1 control cells (Supplemental Figure 1E). Using benzo[a]pyrene (BaP) carcinogen, we generated BaP-4 and DMSO-1 PyMt cell clones following the same procedure described for DMBA/DMSO-treated PyMt clones above (Supplemental Figure 1F). Although BaP-4 tumors initially grew to a palpable size, they were completely rejected in WT C57BL/6 mice by 50 days after injection (Figure 1J). In contrast, BaP-4 cells formed large tumors in CD8^+^ T and CD4^+^ T cell–depleted WT mice (Figure 1K). Vehicle-treated DMSO3-1 and DMSO-1 PyMt cell clones formed tumors in WT mice with growth kinetics comparable to parental PyMt cells (Supplemental Figure 1G). Thus, chemical carcinogen-induced enhancement of cancer cell immunogenicity is a reproducible phenomenon.

### Carcinogen-induced cancer cell immunogenicity is not dependent on increased neoantigen load.

**Figure 2:**
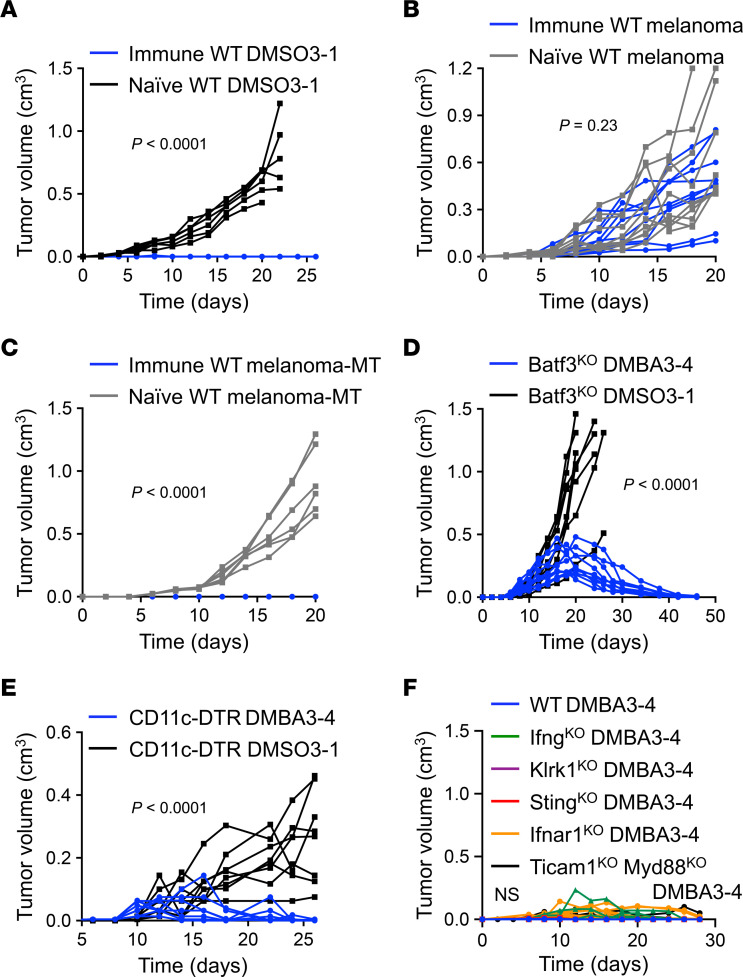
High immunogenicity of DMBA3-4 cells is not associated with increased TMB or neoantigen-directed immunity. (**A**) DMSO3-1 tumor growth in WT mice that previously rejected DMBA3-4 cells (immune, *n* = 10) versus naive WT mice (*n* = 6). (**B**) Melanoma tumor growth in WT mice that previously rejected DMBA3-4 cells (immune, *n* = 9) versus naive WT mice (*n* = 10). (**C**) Melanoma-MT tumor growth in DMBA3-4-immunized (immune, *n* = 10) and naive WT mice (*n* = 6). (**D**) DMBA3-4 and DMSO3-1 tumor growth in Batf3^KO^ mice (*n* = 10 for DMBA3-4 and *n* = 8 for DMSO3-1 group). (**E**) DMBA3-4 and DMSO3-1 tumor growth in diphtheria toxin-treated (DT-treated) CD11c-DTR mice (*n* = 8 per group). (**F**) DMBA3-4 tumor growth in WT (*n* = 10), Ifng^KO^ (*n* = 8), Klrk1^KO^ (*n* = 8), Sting^KO^ (*n* = 12), Ifnar1^KO^ (*n* = 14), and Ticam1^KO^ Myd88^KO^ (*n* = 7) mice. Mice received 100,000 DMBA3-4; 100,000 DMSO3-1; 500,000 melanoma; or 500,000 melanoma-MT cells per injection site. 2-way ANOVA.

The whole exome sequencing (WES) of DMBA3-4 and BaP-4 cells compared with their DMSO-treated controls revealed 237 and 470 mutations, respectively (Supplemental Tables 1 and 2). These mutations mostly covered 3′UTR, 5′UTR, intronic, and noncoding RNA regions of the genome (Supplemental Tables 1 and 2). Surprisingly, the most immunogenic, DMBA3-4 cells lacked missense single nucleotide variants (SNVs) compared with DMSO3-1 cells (Supplemental Table 1). This finding suggests that the high immunogenicity of DMBA3-4 cells cannot be attributed to an increased missense mutation-derived neoantigen load. As predicted from the mutational impact of DMBA and BaP on DNA (16, 17), most of the mutations in DMBA3-4 and BaP-4 cells were single-base C→A transversion (Supplemental Figure 2, A and B). Apart from missense SNVs, chemical carcinogens induced insertion-deletion (InDel) mutations and other translocations/fusions, which may produce immunogenic neoantigens in DMBA3-4 and BaP-4 cells (Supplemental Tables 1 and 2) (18, 19). To examine the role of tumor-specific T cell immunity in the rejection of DMBA3-4 cells experimentally, we injected WT mice that rejected DMBA3-4 cells (i.e., immune mice) with DMSO3-1 cells lacking any neoantigens. The immune mice rejected DMSO3-1, indicating that neoantigen-specific T cells were not required for the rejection phenotype (Figure 2A). However, the immune mice were not able to control the tumor growth of melanoma cells that lacked shared tumor-associated antigens (Figure 2B). The DMBA3-4 and DMSO3-1 cell lines were derived from PyMt cells, which express mouse polyomavirus middle T antigen (MT). Considering that MT can serve as a bona fide antigen, we further investigated whether T cell immunity against DMBA3-4 targets this tumor-associated antigen. A melanoma cell line expressing MT antigen (melanoma-MT) was injected into DMBA-4 immune versus naive WT mice. Notably, the melanoma-MT cells were rejected in DMBA3-4–immune mice while forming tumors in naive mice (Figure 2C). These findings demonstrate that the main immunogenic effect of DMBA in DMBA3-4 is not mediated through neoantigen generation.

To determine the mechanism that activated antitumor T cell immunity against DMBA3-4 cells, we investigated whether the loss of antigen presenting cells (APCs) or the deletion of major innate immune factors implicated in antitumor immunity could block the rejection of DMBA3-4 cells. DMBA3-4 tumor rejection persisted in *Batf3^–/–^* (Batf3^KO^) mice and CD11c^+^ dendritic cell–depleted (DC-depleted) animals (Figure 2, D and E). Macrophage depletion accelerated DMBA3-4 tumor rejection (Supplemental Figure 2C). These findings suggest a redundant function of several APC types to induce T cell immunity against immunogenic DMBA3-4 cells. Next, we examined the role of IFN-γ, NKG2D, STING, type I interferons, Toll-like receptors signaling, and natural killer cells in DMBA3-4 tumor rejection. DMBA3-4 tumor rejection persisted in *Ifng^–/–^* (Ifng^KO^); *Klrk1^–/–^* (Klrk1^KO^); *Sting^–/–^* (Sting^KO^); *Ifnar1^–/–^* (Ifnar1^KO^); *Ticam1^–/–^,Myd88^–/–^* (Ticam1^KO^ Myd88^KO^); and *Ncr1^iCre^,ROSA^DTR^* mice (Figure 2F and Supplemental Figure 2D).

To further evaluate the gain of an immunogenic factor as the cause of carcinogen-exposed cancer cell rejection, we examined the expression of luciferase and mCherry proteins in the cell clones derived from PyMt cells. Single cell selection may lead to differential expression of reporter genes and thus induce differential immunogenicity. DMBA3-4 cells exhibited higher luciferase enzyme activity compared with DMSO3-1 cells; however, BaP-4 cells displayed lower luciferase activity compared with DMSO-1 control cells (Supplemental Figure 3A). Although DMBA3-4 cells had higher mCherry expression compared with DMSO3-1 cells (Supplemental Figure 3B), mCherry overexpression in DMSO3-1 cells (using Lenti-mCherry infection) did not have any impact on DMSO3-1 tumor growth kinetics (Supplemental Figure 3, B and C). These data exclude the possibility that mCherry protein levels mediated the immunogenicity of DMBA3-4 cells. Collectively, these findings indicate that carcinogen-induced cancer immunogenicity may not be driven by the gain of an immunogenic factor.

### Loss of an immunosuppressive TME explains carcinogen-induced cancer immunogenicity.

**Figure 3:**
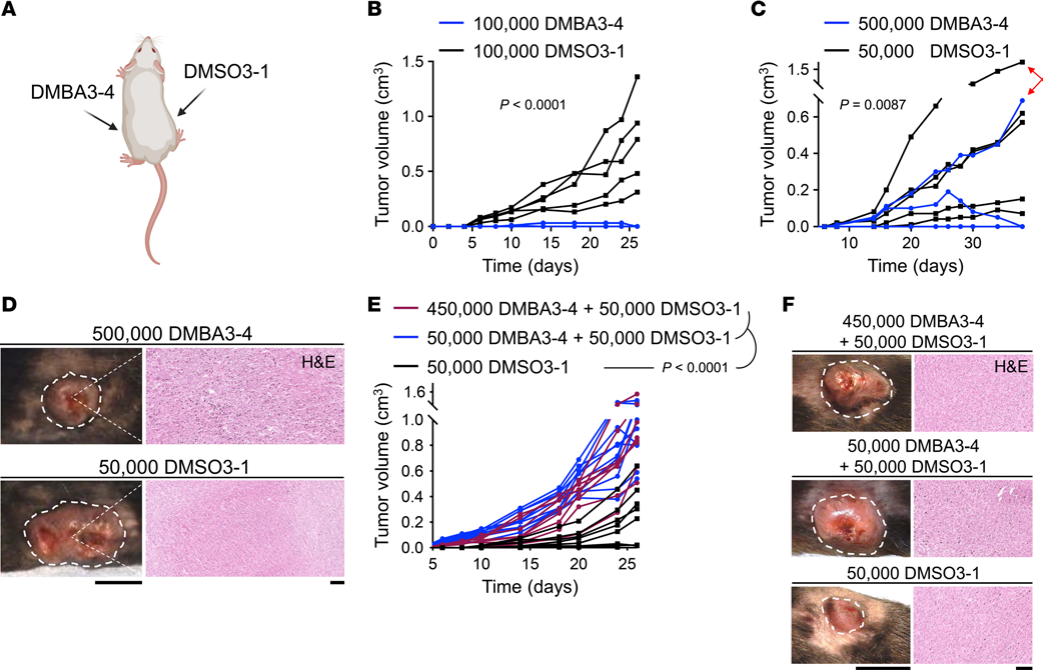
Carcinogen-exposed cancer cells do not form an immunosuppressive TME. (**A**) Schematic illustration of DMBA3-4 and DMSO3-1 cell coinjection into WT mice. (**B**) DMBA3-4 and DMSO3-1 tumor growth simultaneously in WT mice (*n* = 5 per group). 100,000 DMBA3-4 cells (left side) and 100,000 DMSO3-1 cells (right side) were injected into each mouse at the same time. (**C**) DMBA3-4 and DMSO3-1 tumor growth in WT mice injected with 500,000 DMBA3-4 cells (left side) and 50,000 DMSO3-1 cells (right side) (*n* = 5 per group). Red arrows point to a DMBA3-4 tumor that grew out and its contralateral DMSO3-1 tumor in the same WT mouse. (**D**) Macroscopic and H&E-stained histological images of DMBA3-4 (left) and DMSO3-1 (right) tumors in the same WT mouse. Scale bars: 1 cm, mouse; 100 μm, histology. (**E**) DMBA3-4 plus DMSO3-1 mixed tumor compared with DMSO3-1 alone tumor growth in WT mice (*n* = 8 for 450,000 DMBA3-4 plus 50,000 DMSO3-1; *n* = 10 for 50,000 DMBA3-4 plus 50,000 DMSO3-1; and *n* = 8 for 50,000 DMSO3-1 group). (**F**) Representative macroscopic and H&E-stained histological images of DMBA3-4 plus DMSO3-1 mixed and DMSO3-1 alone tumors in WT mice. Scale bars: 1 cm, mouse; 100 μm, histology. 2-way ANOVA.

To investigate whether an immunogenic factor released by DMBA3-4 tumors could lead to the rejection of concurrently developing DMSO3-1 tumors, we coinjected WT mice with DMBA3-4 (left side) and DMSO3-1 cells (right side) (Figure 3A). 100,000 DMBA3-4 cells were rejected while 100,000 DMSO3-1 cells injected contralaterally formed large tumors, as they did in naive WT mice (Figure 3B). To determine whether increasing the ratio of DMBA3-4:DMSO3-1 from 1:1 to 10:1 could increase the likelihood of DMSO3-1 tumor rejection, we coinjected WT mice with 500,000 DMBA3-4 (left side) and 50,000 DMSO3-1 cells (right side). Surprisingly, instead of finding any DMSO3-1 tumor rejection, we discovered that 1 out of 5 DMBA3-4 tumors grew in the WT mice (Figure 3, C and D). This unexpected finding suggested that the presence of DMSO3-1 tumor blocked the rejection of DMBA3-4 tumor in the same mouse. To further examine this concept, we injected WT mice with a mixture of DMBA3-4 + DMSO3-1 cell delivered to the same site. The 50,000 + 50,000 and 450,000 + 50,000 mixtures of DMBA3-4 plus DMSO3-1 cells resulted in consistent tumor formation in WT mice, which grew larger than 50,000 DMSO3-1 cells alone (Figure 3, E and F). Notably, there were no significant differences in the number of CD3^+^ T, CD4^+^ T, and CD8^+^ T cells infiltrating the DMBA3-4 tumor that grew on the contralateral side of DMSO3-1 tumor, DMBA3-4 + DMSO3-1 tumors, and DMSO3-1 tumors (Supplemental Figure 4). These findings indicate that an immunosuppressive environment established by DMSO3-1 cells locally and systemically prevented the rejection of DMBA3-4 cells in WT mice. Thus, the immunogenicity of DMBA3-4 cells is likely driven by the loss of their ability to establish an immunosuppressive TME rather than the gain of an immunogenic factor.

### Carcinogen-exposed cancer cells reprogram immunosuppressive tumor-associated macrophages.

**Figure 4:**
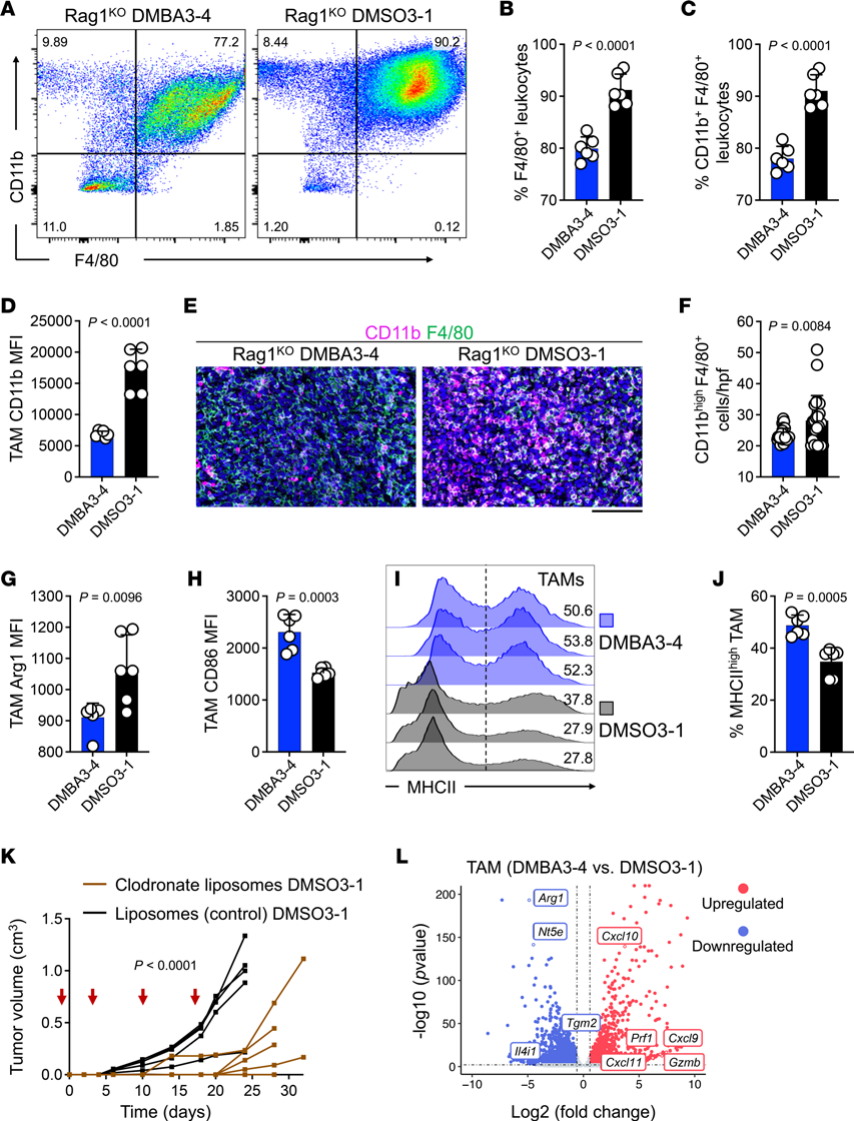
Carcinogen-induced TAMs have antitumor properties. (**A**) Representative flow cytometric analysis of DMBA3-4 and DMSO3-1 TAMs in the tumors from Rag1^KO^ mice. Numbers on the dot plots represent the percent cells within each gate. (**B**) F4/80^+^ leukocyte (TAM) frequencies in DMBA3-4 and DMSO3-1 tumors (*n* = 6 per group). (**C**) CD11b^+^ F4/80^+^ leukocyte (TAM) frequencies in DMBA3-4 and DMSO3-1 tumors (*n* = 6 per group). (**D**) Mean fluorescence intensity (MFI) of CD11b expression on DMBA3-4 and DMSO3-1 TAMs (*n* = 6 per group). (**E**) Representative immunofluorescence images of CD11b- and F4/80-stained DMBA3-4 and DMSO3-1 tumors from Rag1^KO^ mice. Scale bar: 100 μm. (**F**) CD11b^hi^ F4/80^+^ TAM counts in DMBA3-4 and DMSO3-1 tumors. TAMs were quantified in 4 randomly selected high-power field (hpf) images per sample (*n* = 6 per group). Each dot represents a hpf image. (**G** and **H**) MFI of (**G**) arginase 1 (Arg1) and (**H**) CD86 expression in DMBA3-4 and DMSO3-1 TAMs (*n* = 6 per group). (**I**) MHCII expression on CD11b^+^ F4/80^+^ TAMs. Numbers on the flow histograms represent the percent MHCII^hi^ TAMs. (**J**) MHCII^hi^ TAM frequencies in DMBA3-4 and DMSO3-1 tumors from Rag1^KO^ mice (*n* = 6 per group). (**K**) DMSO3-1 tumor growth in WT C57BL/6 mice treated with clodronate liposome versus control liposome. Liposome i.p. injections were performed on days 1, 3, 10, and 17 after tumor inoculation (red arrows, *n* = 6 per group). (**L**) Differentially expressed (DE) genes in DMBA3-4 versus DMSO3-1 TAMs from Rag1^KO^ mice. The significantly upregulated and downregulated genes are indicated with red and blue dots, respectively (*n* = 6 per group). Unpaired *t* test (**B**–**D**, **F**–**H**, and **J**) and 2-way ANOVA (**K**), bar graphs show mean + SD.

To determine which immunosuppressive cell types were reduced in DMBA3-4 tumors, we compared DMBA3-4 and DMSO3-1 tumors that developed in Rag1^KO^ mice. Flow analysis of tumor-derived CD45^+^ leukocytes demonstrated a significant reduction in the frequency of F4/80^+^ and CD11b^+^F4/80^+^ TAMs in DMBA3-4 compared with DMSO3-1 tumors (Figure 4, A–C). Furthermore, TAMs in DMBA3-4 tumors showed lower mean fluorescence intensity (MFI) of CD11b expression compared with TAMs in DMSO3-1 tumors (Figure 4D). Consistently, tissue immunostaining showed fewer CD11b^hi^ TAMs in DMBA3-4 compared with DMSO3-1 TME (Figure 4, E and F). Although the frequency of granulocytes did not differ in DMBA3-4 and DMSO3-1 TME (Supplemental Figure 5, A and B), CD11b MFI was reduced in DMBA3-4 tumor-derived granulocytes (Supplemental Figure 5, C and D).

Further macrophage characterization demonstrated that DMBA3-4 TAMs expressed lower Arginase1 (M2 macrophage marker) but higher CD86 and MHCII (M1 macrophage markers) compared with DMSO3-1 TAMs (Figure 4, G–J). Indeed, the depletion of macrophages in DMSO3-1 tumors suppressed their growth in WT mice, indicating the immunosuppressive nature of TAMs in DMSO3-1 TME (Figure 4K). To further determine the nature of carcinogen-induced TAMs, we compared the transcriptional profiles of DMBA3-4 and DMSO3-1 TAMs from tumors that developed in Rag1^KO^ mice. The proinflammatory genes represented by *Cxcl9, Cxcl10, Cxcl11, Prf1,* and *Gzmb* were found to be significantly enriched in DMBA3-4 TAMs, while classical M2 genes represented by *Arg1, Nt5e, Tgm2,* and *Il4i1* were significantly downregulated in DMBA3-4 compared with DMSO3-1 TAMs (Figure 4L). Gene set enrichment analysis (GSEA) further demonstrated that antigen processing and presentation, phagosome, cytotoxicity, and toll-like receptor signaling pathways were enriched in DMBA3-4 compared with DMSO3-1 TAMs (Supplemental Figure 5E). Thus, compared to classical immunosuppressive TAMs found in breast tumors, TAMs in carcinogen-exposed tumors exhibit heightened antitumor properties.

### Carcinogen exposure downregulates M-CSF and CD155 expression by cancer cells.

**Figure 5:**
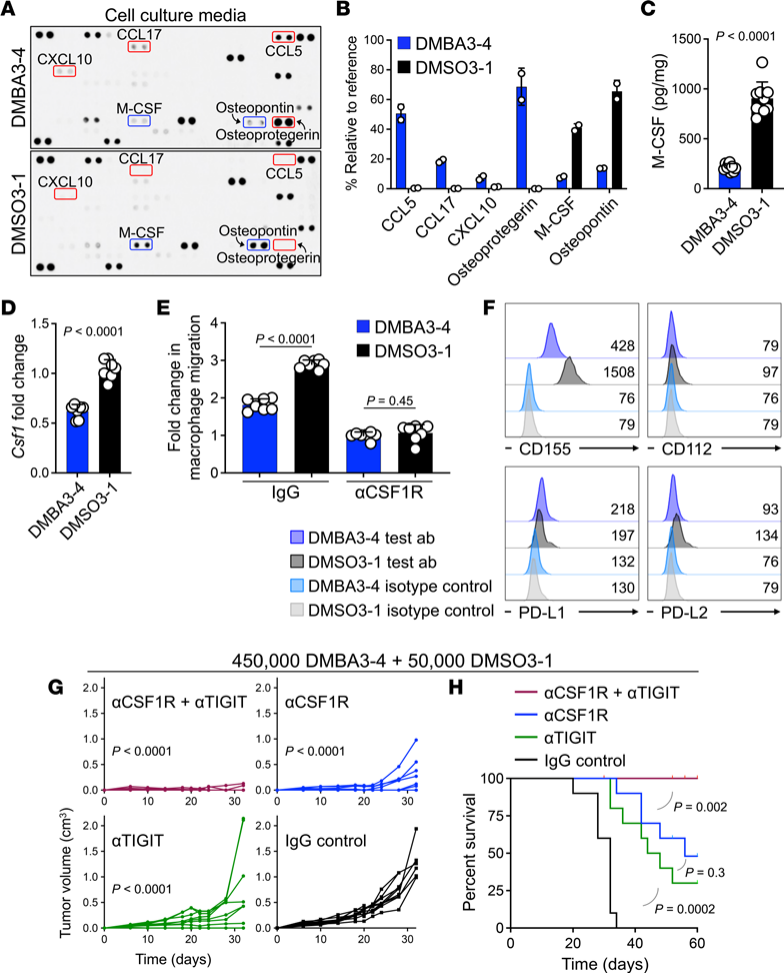
Carcinogen-induced immunogenicity is dependent on reduced M-CSF and CD155 expression by cancer cells. (**A**) Cytokine array on supernatant from DMBA3-4 and DMSO3-1 cells. Red and blue boxes indicate the upregulated and downregulated proteins secreted by DMBA3-4 compared with DMSO3-1 cells, respectively. (**B**) Relative levels of the select upregulated and downregulated proteins from the DMBA3-4/DMSO3-1 cytokine array (n=2 per group). (**C**) M-CSF protein levels in DMBA3-4 compared with DMSO3-1 cell lysates (*n* = 9 per group). (**D**) *Csf1* mRNA expression levels in DMBA3-4 compared with DMSO3-1 cells (*n* = 7 per group). (**E**) BMDM migration toward DMBA3-4 versus DMSO3-1 cells in the presence of anti-CSF1R or IgG control antibody. Fold change is determined as the ratio of BMDM migration in the absence of tumor cells at 96 hours after coculture (n=7 per group). (**F**) CD155, CD112, PD-L1 and PD-L2 expression on DMBA3-4 and DMSO3-1 cells. Numbers on the flow histograms represent the ligands’ MFI. (**G**) DMBA3-4 plus DMSO3-1 mixed tumor growth in WT mice treated with anti-TIGIT and/or anti-CSF1R antibody compared with IgG-treated controls (*n* = 10 per group). Mice received 450,000 DMBA3-4 plus 50,000 DMSO3-1 cells per injection site. (**H**) Survival rate of WT mice that received DMBA3-4 plus DMSO3-1 cells and treated with anti-TIGIT and/or anti-CSF1R antibody compared with IgG-treated controls (*n* = 10 per group). Unpaired *t* test (**C**–**E**), 2-way ANOVA (**G**) and log-rank test (**H**), bar graphs show mean ± SD.

To explore the mechanism by which carcinogen-exposed cancer cells affected TAM development, we compared the secretome profiles of DMBA3-4 and DMSO3-1 cells (Figure 5A and Supplemental Figure 6A). DMBA3-4 cell supernatant showed elevated levels of CCL5, CCL17, CXCL10, and Osteoprotegerin and reduced levels of M-CSF and Osteopontin (OPN) (Figure 5, A and B). CCL5 and CXCL10 are known to be interferon-stimulated genes (ISGs), which can induce T cell immunity (20, 21). However, DMBA3-4 cells were rejected in mice lacking CCL5 and CXCL10 receptors (*Ccr5^–/–^ Cxcr3^–/–^* or Ccr5^KO^ Cxcr3^KO^) and mice lacking type I–IFN receptor (IFNAR) and CXCR3 (*Ifnar1^–/–^ Cxcr3^–/–^* or Ifnar1^KO^ Cxcr3^KO^) (Supplemental Figure 6B). Considering the important role of M-CSF in macrophage recruitment and differentiation, we confirmed M-CSF protein and *Csf1* RNA downregulation in DMBA3-4 compared with DMSO3-1 cells (Figure 5, C and D). To investigate the role of cancer cell-derived M-CSF in TAM recruitment, we cocultured DMBA3-4 and DMSO3-1 cells together with bone marrow–derived macrophages (BMDMs) in a migration assay (Supplemental Figure 6C). Significantly fewer macrophages migrated toward DMBA3-4 cells compared with DMSO3-1 cells (*P* < 0.0001, Figure 5E). Importantly, antibody blockade of M-CSF receptor (αCSF1R) abrogated any differences in macrophage migration toward DMBA3-4 versus DMSO3-1 cells (Figure 5E). Using secretome and gene expression analysis, we found that BaP-4 cells also significantly downregulated M-CSF protein and *Csf1* gene expression levels compared with DMSO-1 control cancer cells (Supplemental Figure 6, D–G). In addition, fibroblast growth factor 21 (FGF-21) and leukemia inhibitory factor (LIF) were markedly downregulated in BaP-4 compared with DMSO-1 cells (Supplemental Figure 6D). However, these proteins were undetectable or not changed in DMBA3-4 versus DMSO3-1 cells (Supplemental Figure 6A).

In addition to secretory factors, we examined the inhibitory ligand expression (PD-L1, PD-L2, CD155,and CD112) on the surface of carcinogen-exposed cancer cells. Interestingly, DMBA3-4 and BaP-4 cells markedly downregulated CD155 expression on their surface compared with DMSO3-1 and DMSO-1 control cells, respectively (Figure 5F and Supplemental Figure 6H). Considering the upregulation of ISGs in DMBA3-4 compared with DMSO3-1 cells, we examined whether ISG suppression by IRF3 siRNA treatment could also affect M-CSF and CD155 expression in DMBA3-4 cells (Supplemental Figure 7, A–C) (22). IRF3 knockdown did not change M-CSF or CD155 expression in DMBA3-4 cells (Supplemental Figure 7, D and E). To determine the functional contribution of M-CSF and CD155 downregulation to carcinogen-induced cancer cell immunogenicity, we examined whether CSF1R and TIGIT blockade could lead to the rejection of DMBA3-4 + DMSO3-1 tumors in WT mice. Anti-CSF1R and anti-TIGIT antibodies alone partially inhibited the growth of DMBA3-4 + DMSO3-1 tumors (Figure 5, G and H). Importantly, the combination of anti-CSFR1 and anti-TIGIT treatment resulted in the complete rejection of 8 out of 10 DMBA3-4 + DMSO3-1 tumors in WT mice (Figure 5, G and H). Thus, carcinogen exposure led to a profound reversal of the immunosuppressive TME by downregulating cancer cell’s expression of M-CSF and CD155.

Next, we investigated whether carcinogen exposure impacted the TME in a spontaneous cancer model. MMTV-PyMt^tg^ mice treated with DMBA at puberty, before any tumor initiation, developed more immunogenic breast tumors compared with MMTV-PyMt^tg^ mice treated with olive oil (carrier control) (Supplemental Figure 8A) (23). DMBA-treated MMTV-PyMt^tg^ mice developed breast tumors with decreased M-CSF expression compared with olive oil–treated MMTV-PyMt^tg^ mice (Supplemental Figure 8B). Furthermore, breast tumors of DMBA-treated MMTV-PyMt^tg^ mice contained significantly fewer immunosuppressive TAMs compared with olive oil–treated animals (Supplemental Figure 8, C and D). These findings indicate that DMBA exposure can also inhibit immunosuppressive TME development in spontaneous breast cancer.

### Carcinogen exposure reprograms TAM differentiation in mouse and human lung cancer.

**Figure 6:**
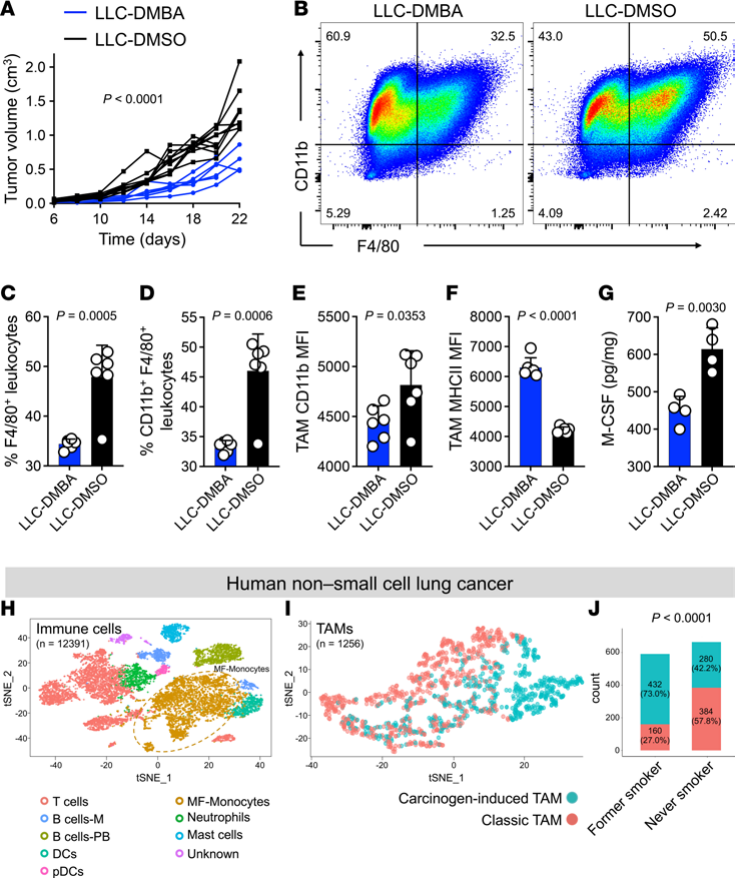
Carcinogen exposure reprograms TAMs in mouse and human lung cancer. (**A**) LLC-DMBA and LLC-DMSO tumor growth in WT mice (*n* = 6 for LLC-DMBA and *n* = 10 for LLC-DMSO group). (**B**) Representative flow cytometric analysis of LLC-DMBA and LLC-DMSO TAMs in the tumors from WT mice. Numbers on the dot plots represent the percent cells within each gate. (**C**) F4/80^+^ leukocyte (TAM) frequencies in LLC-DMBA and LLC-DMSO tumors (*n* = 6 per group). (**D**) CD11b^+^ F4/80^+^ leukocyte (TAM) frequencies in LLC-DMBA and LLC-DMSO tumors (*n* = 6 per group). (**E**) CD11b MFI on LLC-DMBA and LLC-DMSO TAMs (*n* = 6 per group). (**F**) MHCII MFI on LLC-DMBA and LLC-DMSO TAMs (*n* = 6 per group). (**G**) M-CSF protein levels in LLC-DMBA compared with LLC-DMSO cell lysates (*n* = 4 per group). (**H**) t-stochastic neighbor embedding (t-SNE) plot of immune cells (*n* = 12,391) isolated from human non-small cell lung cancer tissues, including B cells: M, memory B cells; B cells-PB, plasmablast; pDCs, plasmacytoid DCs; MF, Monocytes, macrophages and monocytes; unknown, unknown cells from general clustering of all cells. (**I**) Carcinogen-induced TAM (*n* = 712) and classical TAM (*n* = 544) subsets of macrophages (*n* = 1,256) in human non-small cell lung cancer tissues. Carcinogen-induced TAMs are distinguished from classical TAMs by upregulation of *CXCL9*, *CXCL10*, *CXCL11*, *PRF1*, and *GZMB* and downregulation of *ARG1*, *NT5E*, *TGM2,* and *IL4I1* genes as defined in Figure 4L. (**J**) Carcinogen-induced and classical TAM distribution in lung cancers of former smokers versus individuals who have never smoked (never smokers). 2-way ANOVA (**A**), unpaired *t* test (**C**–**G**) and χ^2^ test (**J**), bar graphs show mean + SD.

To further investigate the impact of carcinogen on TAMs differentiation, we treated mouse Lewis lung carcinoma (LLC) cells (24) with DMBA to mimic the effect of smoking on lung cancer (Supplemental Figure 9A) (25). Subcutaneous tumors from DMBA-exposed LLC cells (LLC-DMBA) grew significantly smaller than DMSO-treated (LLC-DMSO) controls in WT mice (Figure 6A). CD8^+^ T + CD4^+^ T cell depletion accelerated the growth of LLC-DMBA tumors in WT mice (Supplemental Figure 9B). Like DMBA3-4 breast cancer cells, markedly fewer F4/80^+^ and CD11b^+^F4/80^+^ TAMs were present in LLC-DMBA compared with LLC-DMSO tumors (Figure 6, B–D). Furthermore, TAMs in LLC-DMBA tumors showed lower CD11b but higher MHCII MFI compared with TAMs in LLC-DMSO tumors (Figure 6, E and F). There was no significant difference in the frequency and CD11b MFI of granulocytes in LLC-DMBA versus LLC-DMSO tumors (Supplemental Figure 9, C–E). Consistent with findings in carcinogen-exposed breast cancer cells, M-CSF protein levels were markedly reduced in LLC-DMBA compared with LLC-DMSO cells (Figure 6G).

To examine the role of carcinogen-induced TAMs in human cancer, we analyzed single-cell RNA–Seq (scRNA-Seq) data obtained from 34 lung cancers in an annotated cohort of former smokers and individuals who had never smoked (NCBI BioProject-PRJNA591860) (26). All immune cells from biopsies and surgical resection (*n* = 12,391) were annotated and clustered into T cells, macrophages/monocytes, B cells, neutrophils, DCs, and mast cells subsets. As expected, macrophages and T cells formed the most abundant immune cell populations in lung cancer (Figure 6H). Macrophages from lung cancers (*n* = 1,256) were reclustered into carcinogen-induced TAM (*n* = 712) versus classical TAM (*n* = 544) groups using the differentially expressed gene set that was defined by comparing DMBA3-4 and DMSO3-1 TAMs (Figure 6I). Carcinogen-induced TAMs were defined as macrophages expressing high levels of human proinflammatory cytokines *CXCL9,* *CXCL10*, *CXCL11*, *PRF1*, and *GZMB*, and classical TAMs were defined as macrophages expressing high levels of *ARG1, NT5E, TGM2,* and *IL4I1* (Figure 4L*)*. Next, we compared the distribution of carcinogen-induced versus classical TAMs among individuals who had never smoked (*n* = 19) and former smokers (*n* = 8). Carcinogen-induced TAMs were significantly enriched in former smokers compared with individuals who had never smoked (432 versus 280 cells), whereas individuals who had never smoked had more classical TAMs compared with former smokers (384 versus 160 cells) (Figure 6J). The studied scRNA-Seq data set was enriched for CD45^+^ immune cells before sequencing library preparation and only 871 cancer cells were identified in the scRNA-Seq data (233 cells from former smokers and 638 from individuals who had never smoked). Nonetheless, *CSF1* (logFC = –0.403, *P* = 0.116) and *PVR* expression (logFC = –0.467, *P* = 0.069) showed lower expression in former smokers compared with individuals who had never smoked. Thus, carcinogen-exposed cancer cells reprograms TAM function in mouse and human lung cancer, which may contribute to the enhanced lung cancer immunogenicity observed in smokers (14).

## Discussion

It is well established that carcinogen exposure, as reflected by increased TMB, enhances cancer immunogenicity and is associated with improved response to immunotherapy (27). The immunogenic impact of carcinogen exposure has largely been attributed to neoantigen generation evoking neoantigen-specific T cell response in cancer (28). By studying a highly immunogenic cancer cell line generated by chemical carcinogen exposure lacking neoantigens our work uncovers neoantigen-independent contributions of carcinogens to cancer immunogenicity. Carcinogen exposure promotes inflammatory cytokine secretion, including CCL5 and CXCL10, by cancer cells. However, the dominant immunological effect of carcinogen exposure relates to its impact on blocking the development of an immunosuppressive TME. This effect, which is partly mediated through the suppression of M-CSF expression by cancer cells, leads to the generation of carcinogen-induced TAMs with antitumor properties. Thus, the immunological consequences of carcinogen exposure extend well beyond increased TMB and neoantigen load and can be extracted and utilized therapeutically to turn cold tumors hot without the risk of exposing cancers to carcinogens or increasing their TMB.

TAMs are a prominent population of immune cells in solid cancers, which are composed of circulating monocyte–derived and tissue-resident macrophages (29). Monocytes can differentiate into classically activated M1 macrophages or alternatively activated M2 macrophages under different conditions (30). GM-CSF, IFN-γ, and lipopolysaccharide-activated (LPS-activated) M1 macrophages stimulate T helper 1 (Th1) response by secreting proinflammatory cytokines, including IL-1β, IL-6, and TNF-α, and recruit Th1 cells through chemokines CXCL9 and CXCL10. M1 macrophages contribute to pathogen clearance and antitumor immunity (31, 32). In contrast, M2 polarization is driven by M-CSF, IL-4, IL-10, and TGF-β. M2 macrophages play important roles in type 2 immune response including wound healing and tissue regeneration (33). M2 macrophages produce antiinflammatory cytokines, including IL-10 and TGF-β, that promote tumor development (34). TAMs can express a combination of M1 and M2 markers, and their polarization is a dynamic process within the TME rather than commonly perceived M2 polarization (35). Considering the highly plastic nature of TAMs, current macrophage-targeting immunotherapies are based on TAM repolarization to increase the ratio of antitumor to protumor macrophages in TME (36). In this study, we identify the carcinogen-induced TAMs, which are defined by increased *Cxcl9*, *Cxcl10*, *Cxcl11*, *Prf1,* and *Gzmb* expression and decreased *Arg1*, *Nt5e*, *Tgm2,* and *Il4i1* expression. Consistent with an antitumor functionality, carcinogen-induced TAMs are enriched for antigen presentation, phagocytosis, cytotoxicity, and Toll-like receptor signaling pathway. Although carcinogen exposure cannot be used to increase patient response to immunotherapy, reprogramming the macrophages to a carcinogen-induced profile (e.g., M-CSF/CSF1R blockade) provide an innovative strategy for boosting antitumor immunity in cold tumors. Besides M-CSF, OPN is significantly reduced in DMBA-treated cancer cells. OPN produced by tumor cells can support their survival in circulation, while tumor- and myeloid cell-derived OPN can render the metastatic tumor more immunosuppressive (37). Thus, decreased OPN expression in carcinogen-exposed cancer cells may contribute to elimination of the immunosuppressive TME.

The limited efficacy of ICB therapy has spurred the investigation of immunosuppressive cells, including classical TAMs, myeloid-derived suppressor cells (MDSCs), and regulatory T cells (Tregs) as immunotherapeutic targets in recent years (38, 39). Immunosuppressive cells play an important role in tumor progression by inhibiting the functions of effector T cells, natural killer cells, and APCs (40). Immunotherapeutic strategies have been developed to reshape the TME, including the inhibition of immunosuppressive cell recruitment to the TME, depletion of immunosuppressive cells in the TME, as well as reprogramming of immunosuppressive cells (41, 42). Among them, an anti-CD33 antibody (Gemtuzumab) targeting MDSCs is shown to restore T cell immunity and improve cancer immunotherapy by depleting CD33-expressing MDSCs (43, 44). Consistent with our findings, M-CSF/CSF1R blockade is found to synergize with immunotherapy and radiotherapy by reducing TAM numbers and inducing TAM repolarization (45, 46). Several clinical trials targeting immunosuppressive cells in cancer have also shown beneficial effects by improving antitumor immunity (47, 48).

We have demonstrated the abundance of carcinogen-induced TAMs in lung cancer of smokers with high cancer immunogenicity compared with classical TAMs dominating the microenvironment of lung cancer in nonsmokers. This finding supports the notion that carcinogen exposure not only enhances T cell immunity by inducing neoantigens in tumors, but also alters TAM differentiation, which enhances tumor immunogenicity. As such, TAM-targeting intervention may synergize with T cell-directed cancer immunotherapies, especially for cancers with high TMB that fail to respond to ICB therapy. This is evident in the rejection of carcinogen-exposed DMBA3-4 cells mixed with control cancer cells, which is achieved by anti-CSF1R and anti-TIGIT combination treatment. Nonetheless, the precise contributions of the immunostimulatory factors to high immunogenicity of DMBA3-4 cells remains to be determined. In fact, we have previously demonstrated that DMBA exposure before cancer initiation leads to the development of immunogenetic spontaneous breast tumors, at least in part, due to the induction of CCL21 expression in tumor cells (23). Future studies are warranted to determine the precise composition of the immune activating and suppressing factors that are sufficient to transform nonimmunogenic tumor cells to be rejected by T cells.

Taken together, our findings demonstrate that the modulation of TAMs and the inhibition of the immunosuppressive TME created by cancer cells contribute to the enhanced cancer immunogenicity associated with carcinogen exposure. Targeting carcinogen-regulated immune factors like M-CSF/CSF1R in combination with ICB therapy highlight promising therapeutic strategies that can be uncovered by exploring the TMB/neoantigen-independent immunological impacts of environmental carcinogens on cancer development.

## Methods

### Mice.

WT C57BL/6 and BALB/c mice were purchased from Charles River Laboratories. Rag1^KO^, Ifng^KO^, Ifnar1^KO^, Klrk1^KO^, Sting^KO^, Batf3^KO^, Ticam1^KO^, Myd88^KO^, Ccr5^KO^, Cxcr3^KO^, CD11c-DTR, and ROSA^DTR^ C57BL/6 mice were purchased from the Jackson Laboratory. Single-KO mice were bred to generate double-KO mice, including Ticam1^KO^ Myd88^KO^, Ccr5^KO^ Cxcr3^KO^, and Ifnar1^KO^ Cxcr3^KO^. Ncr1^iCre^ mice (a gift from Eric Vivier, Aix Marseille University Hospital, Marseille, France) were bred with ROSA^DTR^ mice to generate Ncr1^iCre^ ROSA^DTR^ mice. MMTV-PyMt^tg^ mice (a gift from David DeNardo, Washington University, St. Louis, Missouri, USA) were maintained on BALB/c background. All mice were housed in pathogen-free facilities in accordance with the guidelines instituted by the animal study committees of Massachusetts General Hospital.

### Cell lines.

The parental MMTV-PyMt-mCherry-Luc (PyMt) cell line was derived from mCherry/luciferase labeling of PyMt-B6 cells, which were derived from a mammary tumor of an MMTV-PyMt transgenic mouse on the C57BL/6 background (15, 23). PyMt cells were treated with 20 μmol/L 7,12-Dimethylbenz[a]anthracene (DMBA, catalog no. D3254, Sigma-Aldrich) or Benzo[a]pyrene (BaP, catalog no. B1760, Sigma-Aldrich) dissolved in DMSO at 30%–50% confluency for 24 hours. Cells were passaged and retreated with DMBA or BaP for an additional 24 hours cycle. DMSO was used as vehicle control. In total, cells were treated with a chemical carcinogen for 3 or 6 cycles before single-cell sorting using fluorescence-activated cell sorter (SH800, Sony). 288 pairs of Zombie-green–negative live single cells were sorted into 3 96-well plates for both carcinogen and vehicle groups. For the first selection, 12 clones in each group were selected for subculture from those that had clonal expansion from a single cell. For the second selection, 6 pairs of clones were selected based on their comparable growth rates in culture plates. For the third selection, the precise growth rates of the 6 clone pairs were further compared using CellTiter 96 nonradioactive cell proliferation assay (catalog no. PAG4000, Promega) and a matched pair was selected for follow-up studies. DMSO3-1 cell line expressing enhanced mCherry (DMSO3-1-mCherry) was generated by Lenti-mCherry transduction and selected with high levels of mCherry on flow cytometry. Mouse 4T1 breast cancer cell line, mouse LLC cells were obtained from ATCC. 4T1 cells were treated with DMBA for 6 rounds and single clones were selected in the same way as PyMt cells. LLC cells were treated with DMBA for 3 rounds, and a pool of treated cells were used in experiments. The D4M.3A.3 melanoma cell line was a gift from David Fisher, Massachusetts General Hospital (49, 50). D4M.3A.3-expressing polyomavirus middle T antigen (melanoma-MT) was constructed by lentivirus-MT infection.

### In vivo tumor engraftment.

Unless otherwise specified, 100,000 breast cancer cells were injected s.c. adjacent to the inguinal mammary fat pad of female WT, Rag1^KO^, Batf3^KO^, Ifnar1^KO^, Sting^KO^, Ticam1^KO^ Myd88^KO^, Ccr5^KO^ Cxcr3^KO^, Ifnar1^KO^, and Cxcr3 ^KO^ mice on the C57BL/6 background. 500,000 D4M.3A.3 melanoma and melanoma-MT cells were injected subcutaneously into the flanks of DMBA3-4-immunized and naive mice on the C57BL/6 background. In the LLC tumor model, 1,000,000 LLC cells were injected s.c. into flanks of naive mice on the C57BL/6 background. For macrophage and T cell depletion, clodronate liposome (catalog no. PBS-02, Liposoma BV), anti-mouse CD4 antibody (catalog no. BE0003, Bio X Cell), and anti-mouse CD8β antibody (catalog no. BE0223, Bio X Cell) were i.p. injected once a week, respectively. For natural killer cell depletion, Ncr1^iCre^, ROSA^DTR^ mice were treated with diphtheria toxin (catalog no. D0564, Sigma-Aldrich) as well as anti-NK1.1 antibody (catalog no. BE0036, Bio X Cell). For DC depletion, CD11c-DTR mice were treated with diphtheria toxin. Tumor growth was monitored daily and measured on regular intervals. When a tumor reached 2 cm in diameter or became ulcerated, it was deemed terminal. In the lung metastasis mouse model, 500,000 DMBA3-4 versus DMSO3-1 cells were i.v. injected into tail vein of WT mice on the C57BL/6 background.

### RNA-Seq and GSEA.

CD45^+^ CD11b^+^ F4/80^+^ TAMs were sorted using SH800S sorter (Sony). Cell lysates were prepared in 5 μL buffer TCL (catalog no. 1031576, Qiagen) + 1% β-mercaptoethanol (catalog no. 21985-023, Thermo Fisher Scientific). Samples were sent to Broad Genomic Service, and the SmartSeq2 platform was used to generate RNA-Seq data. Differentially expressed genes were determined using Limma package (51) in R. GSEA was performed using GSEA software and plotted by ClusterProfiler (52) and ggplot2 packages in R. RNA-Seq data were analyzed for gene set enrichment in DMBA3-4-TAMs compared with DMSO3-1-TAMs. Original data are available as the National Center for Biotechnology Information (NCBI) Gene Expression Omnibus database (GSE237536).

### Exome sequencing and analysis.

Genomic DNA (gDNA) was extracted from cultured cell lines with DNeasy Blood and Tissue Kit (catalog no. 69504, Qiagen). The exomes of gDNA were sequenced by Novogen. Exons were captured using magnetic beads and then enriched for library preparation. Sequencing was performed on an Illumina platform. Somatic SNVs and InDels were detected and filtered with the tools muTect (53) and Strelka (54). Accumulated somatic mutations in carcinogen-exposed cell lines were analyzed by comparison to its vehicle control–exposed pair sample. Original data are available as NCBI BioProjects #PRJNA860919 and #PRJNA861664.

### Histology and immunofluorescence.

Tumors, lungs, and skin around tumor cell injection site were harvested, fixed with 4% paraformaldehyde (Sigma-Aldrich), and further embedded in paraffin. Then, 5 μm sections were cut from paraffin-embedded tissues samples for H&E and immunofluorescence staining. For tumor infiltrating T cell staining, sections were incubated with CD3 and CD8α or CD4 primary antibodies followed by Alexa Flour-647-labeled goat anti-rat IgG (catalog no. A21247, Thermo Fisher Scientific) and Alexa Flour-488-labeled goat anti-Rabbit IgG (catalog no. A11034, Thermo Fisher Scientific)**)**. For tumor associated–macrophage staining, sections were incubated with rabbit anti-mouse F4/80 followed by poly HRP-conjugated secondary antibody and Alexa Fluor tyramide reagent in an Alexa Fluor 488 Tyramide SuperBoost Kit (catalog no. B40943, Thermo Fisher Scientific). The primary-secondary-HRP complex were removed with AR6 buffer (catalog no. AR6001KT, Perkin Elmer) by microwave treatment before rabbit anti-mouse CD11b primary antibody and Alexa Fluor 647 goat anti-rabbit secondary antibody incubation. The primary antibodies used in immunofluorescence staining are listed in Supplemental Table 3. The immunofluorescence staining sections were counterstained with DAPI nuclear stain (catalog no. D3571, Thermo Fisher Scientific) and mounted in Prolong GOLD antifade solution (catalog no. P36930, Invitrogen). Images were scanned with AxioScan (Zeiss) and analyzed with the Zeiss ZEN Image Processing software. Quantification of cell population was performed with HALO Image Analysis Platform (Indica Labs).

### Protein analysis.

Cell culture supernatants and cell lysates were collected for protein analysis. An equal volume of cell supernatants were harvested when cells reached 90% confluency and concentrated 300-fold with the SpeedVac Vacuum Concentrator (SPD1010, Thermo Fisher Scientific). The expression of cytokines and chemokines in the cell supernatants were determined with Proteome Profiler Mouse XL Cytokine Array (catalog no. ARY028, R&D Systems) according to the manufacturer’s protocol. Array images were scanned by scanner (Epson) and spot intensity was analyzed with Protein Array Analyzer for Image J. The expression of IFN-α and IFN-β in cell supernatants was determined with IFN-α (catalog no. BMS6027TWO, Thermo Fisher Scientific) and IFN-β (catalog no. 439407, Biolegend) ELISA kits. Cell lysates were prepared for further validation of M-CSF expression. Cells were harvested and lysed with RIPA lysis buffer (catalog no. 89900, Thermo Fisher Scientific) supplemented with 4% protease inhibitor (catalog no. A32955, Thermo Fisher Scientific). Total protein concentration of cell lysates was quantified using Pierce BCA Protein Assay Kit (catalog no. 23225, Thermo Fisher Scientific). The expression of M-CSF in cell lysates was measured with the ELISA kit for mouse M-CSF (catalog no. EMCSF1, Thermo Fisher Scientific) following the manufacturer’s instructions. The relative M-CSF concentration was determined by M-CSF concentration/total protein concentration.

### Lentivirus packaging and transduction.

The plasmid pLV-mCherry (catalog no.36084, Addgene) was cotransfected with the packaging plasmids pCMV Δ R8.2 (catalog no.12263, Addgene) and pMD2.G (catalog no.12259, Addgene) into 293T cells to package lentivirus Lenti-mCherry. The plasmid pCDH-3xFLAG-GFP-puroR (catalog no.167463, Addgene) was inserted into cloned mouse polyomavirus middle T antigen transcripts before cotransfection with pMDLg/pRRE (catalog no.12251, Addgene) and pRSV-Rev (catalog no.12253, Addgene) in 293T cells to package Lenti-MT. The viral supernatant was harvested at 48 and 72 hours after transfection. After centrifuging at approximately 500*g* for 5 minutes to pellet any packaging cells and filtering through a 0.45 μm PES filter, the virus supernatant was used for the transduction of target cells. DMSO3-1-mCherry cells was selected using fluorescence-based screening, while melanoma-MT cells were selected using puromycin selection. The cells were subsequently screened to confirm the expression of the target protein.

### Luciferase activity assay.

Luciferase activity was assessed using the Luciferase Assay System (catalog no. G7941, Promega) following the manufacturer’s instructions. A total of 50,000 DMBA3-4, DMSO3-1, BaP-4, and DMSO-1 cells were seeded in a 96-well plate and incubated at 37°C overnight. The assay plates were then removed from the incubator and allowed to equilibrate at room temperature (22–25°C) for 15 minutes. 100 μL of Bio-Glo Reagent was added to each well of the assay plate. The plate was subsequently incubated at room temperature for 15 minutes. Luminescence was measured using a Synergy Neo2 luminescence microplate reader (Biotek).

### Spontaneous breast carcinogenesis studies.

MMTV-PyMt^tg^ female mice received 1 mg of DMBA dissolved in 100 μL of olive oil or 100 μL of olive oil (carrier alone) by oral gavage at 4–6 weeks of age. Tumor onset and growth were monitored every week. The animals were harvested once a tumor reached 2 cm in diameter or the mice showed any sign of distress or weight loss.

### Quantitative PCR.

The mouse *Csf1*, *Ifnb1*, *Oas2,* and *Isg15* mRNA expression was determined by quantitative PCR using iTaq Universal SYBR Green Supermix (catalog no.1725121, Bio-Rad) on ABI 7500 PCR system (Thermo Fisher Scientific). The primer pairs are listed in Supplemental Table 4. All primers shown are 5′ to 3′.

### Flow cytometry.

Tumor single-cell suspensions were prepared after Collagenase IV (catalog no. LS004189, Worthington Biochemical) and DNase I (catalog no. M0303S, New England Biolabs) digestion and tumor infiltrating immune cells were enriched with CD45 MicroBeads (catalog no. 130-052-301, Miltenyi Biotec) on magnetic columns (Miltenyi Biotec). Cells were stained with the following surface marker antibodies: anti-CD45, anti-CD11b, anti-F4/80, anti-MHCII, anti-CD206, and anti-CD86 (Supplemental Table 3) and fixed and permeabilized by True-Nuclear Transcription Factor Buffer Set (catalog no.424401, Biolegend) for anti-Arginase 1 antibody staining (Supplemental Table 3). For inhibitory ligand staining, cultured cell lines were collected by trypsin digestion and stained with inhibitory ligand antibodies: anti-CD155, anti-CD112, anti-PD-L1, and anti-PD-L2 (Supplemental Table 3). Stained cells were detected by a LSR Fortessa X-20 flow cytometer (BD Bioscience) and data were analyzed with FlowJo software (Tree Star).

### BMDMs chemotaxis assay.

Bone marrow cell suspensions were collected by flushing femurs and tibias of WT C57BL/6 mice (Charles River) and cultured in completed DMEM with 20 ng/mL mouse recombinant M-CSF (catalog no.576406, Biolegend). Fully differentially BMDMs were collected at Day 7 for chemotaxis assay. 10,000 BMDMs in 100 μL/well R10 medium were added onto the top chamber of a 12 well Transwell plate (catalog no.3421, Corning) and 600 μL/well of 20,000 DMBA3-4 or DMSO3-1 cells containing blocking antibody for mouse CSF1R at 20 μg/mL (catalog no.BE0213, BioXCell) or IgG control (catalog no.BE0289, BioXCell) were loaded in the bottom chamber. Migrated cells were collected from the bottom chamber after 96 hours for flow analysis. The average MFI of CD11b and F4/80 were analyzed for CD45^+^ cells, and the migrated numbers were determined by the ratio of CD45^+^CD11b^+^F4/80^+^ cells to absolute counting beads (catalog no.C36950, Thermo Fisher Scientific).

### Macrophage and cancer cell analysis from lung cancer scRNA-Seq data.

scRNA-Seq data of non-small cell lung cancer were downloaded from https://drive.google.com/drive/folders/1sDzO0WOD4rnGC7QfTKwdcQTx3L36PFwX?usp=sharing All code used to generate the results of this study were downloaded from https://github.com/czbiohub/scell_lung_adenocarcinoma (commit ID: de138c79bcfc2fa3a28c8a039a28ab560da78099.). Standard procedures for filtering, variable gene selection, dimensionality reduction, and clustering were performed using the Seurat v3 in RStudio (55) using R 3.6.0, where cells with fewer than 500 genes and 50,000 reads were excluded. We used DoubletFinder (56) to identify potentially sorted doublet cells. Variable genes (Ngenes = 2,000) were selected using a threshold for dispersion, with z-scores normalized by expression level. The variable genes were projected onto a low-dimensional subspace using principal component analysis. Cells were visualized using a 2-dimensional tSNE on the same distance metric. All cells annotated as immune cells (*n* = 12,391) were clustered using the following parameters (Ngenes = 2,000, Npc = 20, Res = 0.7). Macrophages (*n* = 1,256) from lung biopsies were further clustered into classical and carcinogen induced TAM subsets using the following parameters (Ngenes = 2,000, Npc = 3, res = 0.2). The difference in the distribution of classical TAMs and carcinogen-induced TAMs in smokers and individuals who had never smoked were analyzed by χ^2^ test. We analyzed differentially expressed genes of lung cancer cells (*n* = 871) using DESeq2. *CSF1* and *PVR* gene expression in smokers and individuals who had never smoked were analyzed by χ^2^ test.

### Genotyping.

PCR was used to genotype genetically engineered mice. Primer pairs used in this study are listed in Supplemental Table 4. All primers shown are 5′ to 3′.

### Statistics.

Graphs and statistical analysis were performed with GraphPad Prism 9. Log-rank tests were used to compare animal survival. A 2-way ANOVA with Šidák’s multiple comparison test was used to compare tumor growth over time between different groups. A 2-tailed unpaired *t* test was used to compare lung metastasis counts, TAMs, T cell, granulocyte quantification, CD11b, CD86, and Arg1 MFI on TAMs and M-CSF expression between test and control groups. 1-way ANOVA with Tukey’s multiple comparison was used to examine the differences in the mean ranks among 3 possible pairwise comparisons. RNA-Seq, exome-Seq and scRNA-Seq analysis were plotted using RStudio. χ^2^ test was used to compare the distribution of classical and carcinogen-induced TAMs between former smokers and individuals who had never smoked. A *P* value of less than 0.05 was considered significant. All error bars represent SD.

### Study approval.

All animal studies were reviewed and approved by the Massachusetts General Hospital Institutional Animal Care and Use Committee.

### Data availability.

The exome sequencing data can be accessed from NCBI database, SRA accession no.: PRJNA860919 and PRJNA861664. The RNA-Seq data can be accessed from NCBI database, GEO accession no: GSE237536. Values for all data points found in graphs can be found in the supplemental Supporting Data Values file. Additional data related to this paper may be requested from the corresponding author.

## Author contributions

SD conceived the study. MH, YX, KL, and SD designed the experiments. MH, YX, KL, FS, ZF, TL, and MA performed the experiments and analyzed the data. MH, YX, and SD interpreted the data. MH and SD wrote the manuscript. The order of the co–first authors was assigned based on their efforts and contributions to the study.

## Supplementary Material

Supplemental data

Supporting data values

## Version 1 10/16/2023

Electronic publication
