# Supplementary material for: Carcinogen exposure enhances cancer immunogenicity by blocking the development of an immunosuppressive tumor microenvironment
Source: J Clin Invest. 2023 Oct 16;133(20):e166494. doi: 10.1172/JCI166494 (PMC10575722; doi:10.1172/JCI166494)
Supplement: Supplemental data [file jci-133-166494-s031.pdf]

Supplemental Materials for

Carcinogen exposure enhances cancer immunogenicity by blocking the development of an immunosuppressive tumor microenvironment

Mei Huang[1], Yun Xia[1], Kaiwen Li[1], Feng Shao[2], Zhaoyi Feng[1], Tiancheng Li[1], Marjan Azin[1], Shadmehr Demehri[1]

[1]Center for Cancer Immunology and Cutaneous Biology Research Center, Department of Dermatology and Center for Cancer Research, Massachusetts General Hospital and Harvard Medical School, Boston, MA 02114, USA.

[2] Department of General Surgery, The First Affiliated Hospital of USTC, Division of Life Sciences and Medicine, University of Science and Technology of China, Hefei, Anhui 230001, China.

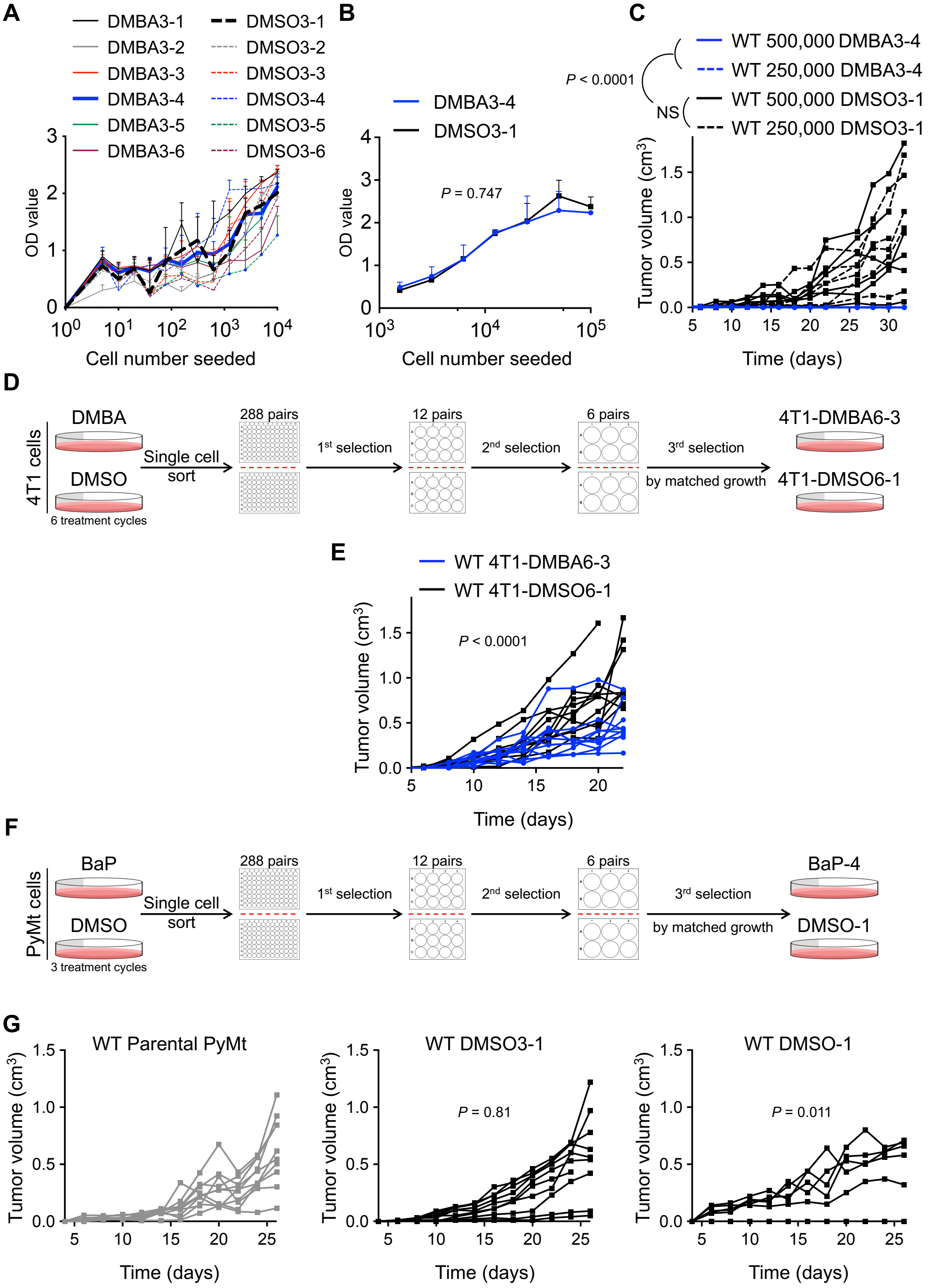
A
DMBA3-1
DMBA3-2
DMBA3-3
DMBA3-4
DMBA3-5
DMBA3-6
DMSO3-1
DMSO3-2
DMSO3-3
DMSO3-4
DMSO3-5
DMSO3-6
OD value
Cell number seeded
B
DMBA3-4
DMSO3-1
P = 0.747
OD value
Cell number seeded
C
WT 500,000 DMBA3-4
WT 250,000 DMBA3-4
WT 500,000 DMSO3-1
WT 250,000 DMSO3-1
P < 0.0001
NS
Tumor volume (cm3)
Time (days)
D
4T1 cells
DMBA
DMSO
6 treatment cycles
Single cell sort
288 pairs
1st selection
12 pairs
2nd selection
6 pairs
3rd selection by matched growth
4T1-DMBA6-3
4T1-DMSO6-1
E
WT 4T1-DMBA6-3
WT 4T1-DMSO6-1
P < 0.0001
Tumor volume (cm3)
Time (days)
F
PyMt cells
BaP
DMSO
3 treatment cycles
Single cell sort
288 pairs
1st selection
12 pairs
2nd selection
6 pairs
3rd selection by matched growth
BaP-4
DMSO-1
G
WT Parental PyMt
WT DMSO3-1
P = 0.81
WT DMSO-1
P = 0.011
Tumor volume (cm3)
Time (days)

Supplemental Figure 1. Generation of carcinogen-exposed breast cancer cells and examination of their growth kinetics *in vitro* and *in vivo*.

(**A**) Proliferation assay for the 6 pairs of DMBA- versus DMSO-treated PyMt cell clones *in vitro*. Cells were serially diluted and seeded for MTT assay (OD: optical density). (**B**) Proliferation assay for DMBA3-4 and DMSO3-1 cells (n=3 per group). (**C**) DMBA3-4 and DMSO3-1 tumor growth after injection of 250,000 and 500,000 cells into WT C57BL/6 mice (n=8 for 500,000 DMBA3-4, n=8 for 250,000 DMBA3-4, n=6 for 500,000 DMSO3-1 and n=6 for 250,000 DMSO3-1 group). (**D**) Schematic diagram of 4T1-DMBA6-3 and 4T1-DMSO6-1 cell clones derived from DMBA and DMSO (vehicle control) exposed 4T1 cell line, respectively. (**E**) 4T1-DMBA6-3 and 4T1-DMSO6-1 tumor growth in syngeneic WT BALB/c mice (n=10 per group). Mice received 100,000 cells per injection site. (**F**) Schematic diagram of BaP-4 and DMSO-1 cell clones derived from BaP and DMSO (vehicle control) exposed PyMt cell line, respectively. Note that BaP-4 and DMSO-1 cells have similar proliferation rates *in vitro*. (**G**) Parental PyMt, DMSO3-1 and DMSO-1 tumor growth in WT C57BL/6 mice (n=10 for parental PyMt, n=10 for DMSO3-1 and n=6 for DMSO-1 group). Mice received 100,000 cells per injection site. DMSO3-1 and DMSO-1 tumor growth data are also shown in Figure 1. Two-way ANOVA.

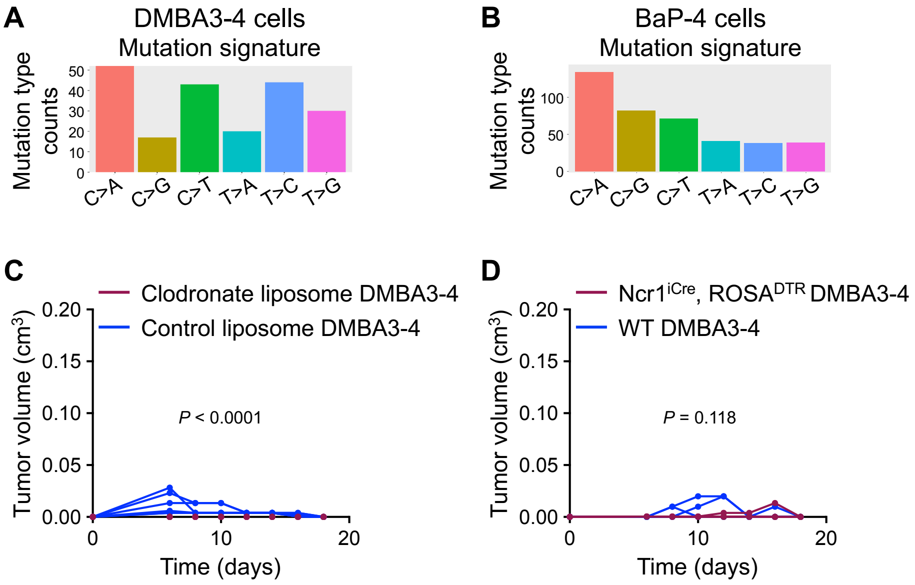

Supplemental Figure 2. Carcinogen-exposed cancer cell mutations and growth in immunodeficient mice.

(**A**) Somatic mutations count in DMBA3-4 cells categorized based on base substitution types. (**B**) Somatic mutations count in BaP-4 cells categorized based on base substitution types. (**C**) DMBA3-4 tumor growth in WT mice treated with clodronate liposome or control liposome (n=6 per group). (**D**) DMBA3-4 tumor growth in Ncr1^{iCre}, ROSA^{DTR} versus WT mice treated with DT (n=4 per group). Mice received 100,000 cancer cells per injection site. Two-way ANOVA.

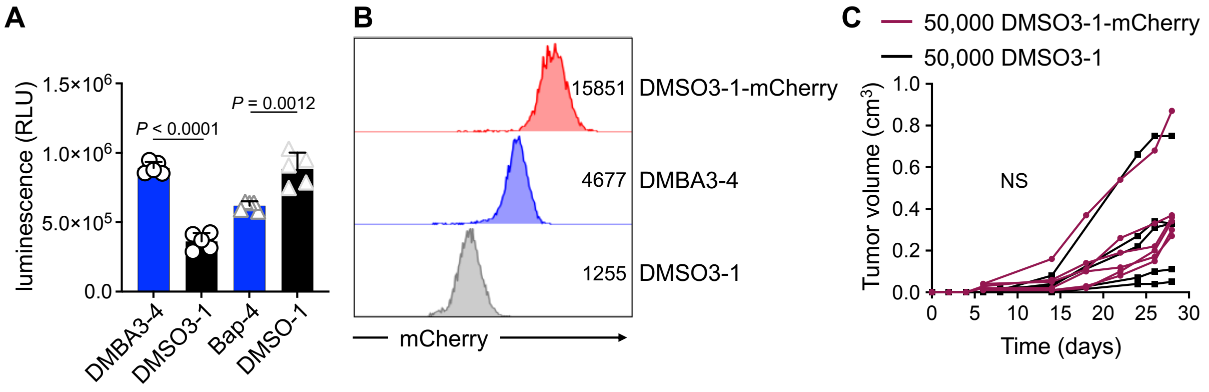

Supplemental Figure 3. Luciferase and mCherry effect on breast tumor immunogenicity. (**A**) The luciferase enzyme activity levels in DMBA3-4, DMSO3-1, BaP-4 and DMSO-1 cells (n=5 per group, bar graphs show mean + s.d., unpaired *t*-test). (**B**) mCherry expression in DMBA3-4, DMSO3-1 and DMSO3-1-mCherry cells. Numbers on the flow histograms represent mCherry MFI. (**C**) DMSO-1-mCherry (test, n=6) and DMSO3-1 (control, n=5) tumor growth in syngeneic WT C57BL/6 mice. NS: not significant, two-way ANOVA.

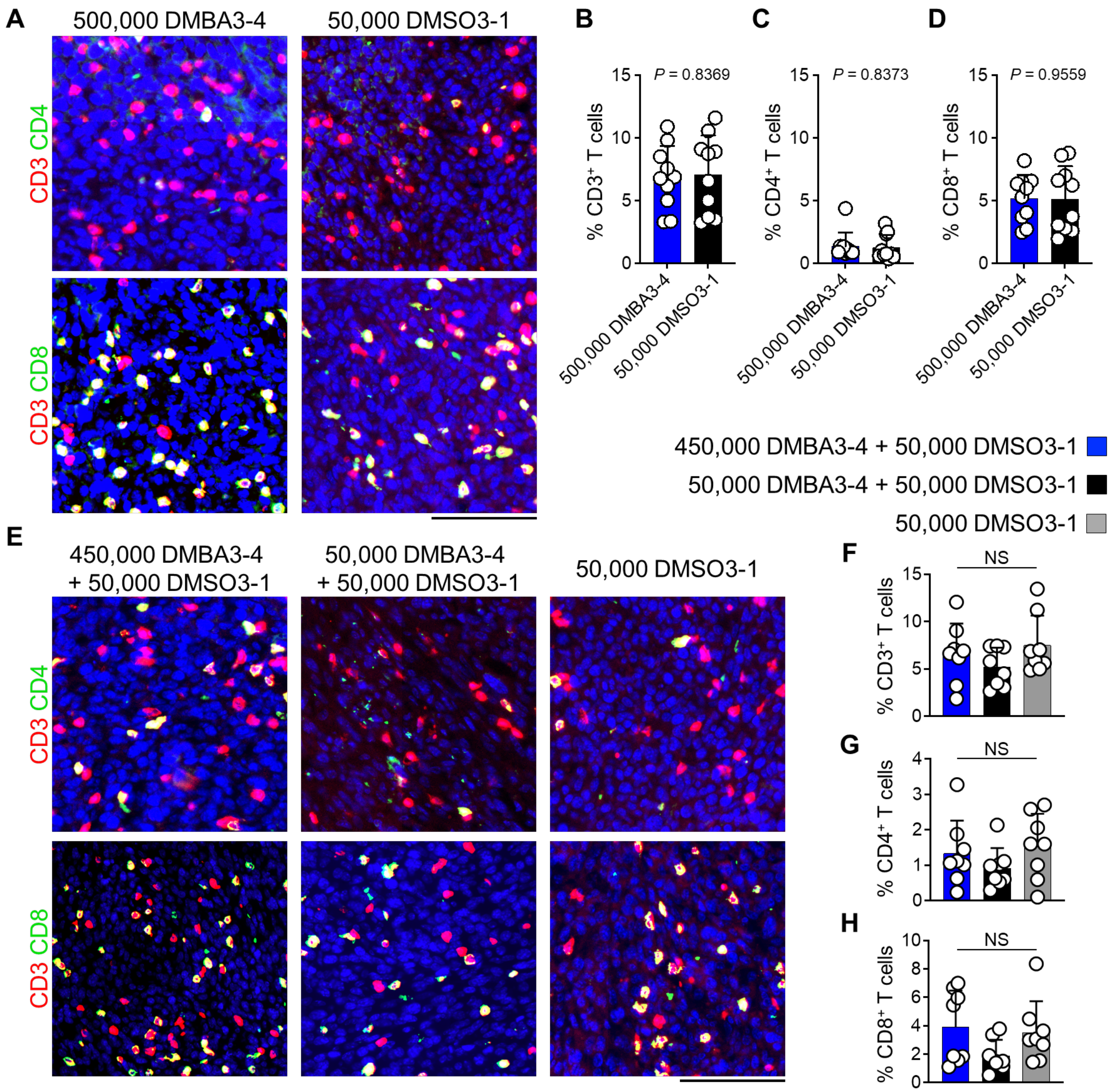

Supplemental Figure 4. T cell quantification in DMBA3-4 tumors that developed in WT mice. (**A**) Representative immunofluorescence (IF) images of CD3/CD4- and CD3/CD8-stained DMBA3-4 and DMSO3-1 tumors that grew in a WT mouse highlighted in Figure 3C (scale bar: 100 µm). (**B**-**D**) Quantification of (B) $CD3^+$ T cells, (C) $CD4^+$ T cells and (D) $CD8^+$ T cells in DMBA3-4 and DMSO3-1 tumors in a WT mouse highlighted in Figure 3C. T cells were counted as percent $DAPI^+$ cells in 10 randomly selected high-power field (hpf) images of each tumor. Each dot represents a hpf image (unpaired *t*-test). (**E**) Representative IF images of CD3/CD4- and

CD3/CD8-stained DMBA3-4 plus DMSO3-1 mixed and DMSO3-1 alone tumors in WT mice (scale bar: 100 μm). (**F**-**H**) Quantification of (F) $CD3^+$ T cells, (G) $CD4^+$ T cells and (H) $CD8^+$ T cells in DMBA3-4 plus DMSO3-1 mixed versus DMSO3-1 alone tumors in WT mice. T cells were counted as percent $DAPI^+$ cells and averaged across 10 randomly selected hpf images per tumor. Each dot represents a tumor (n=8 per group, NS: not significant, one-way ANOVA). Bar graphs show mean + s.d.

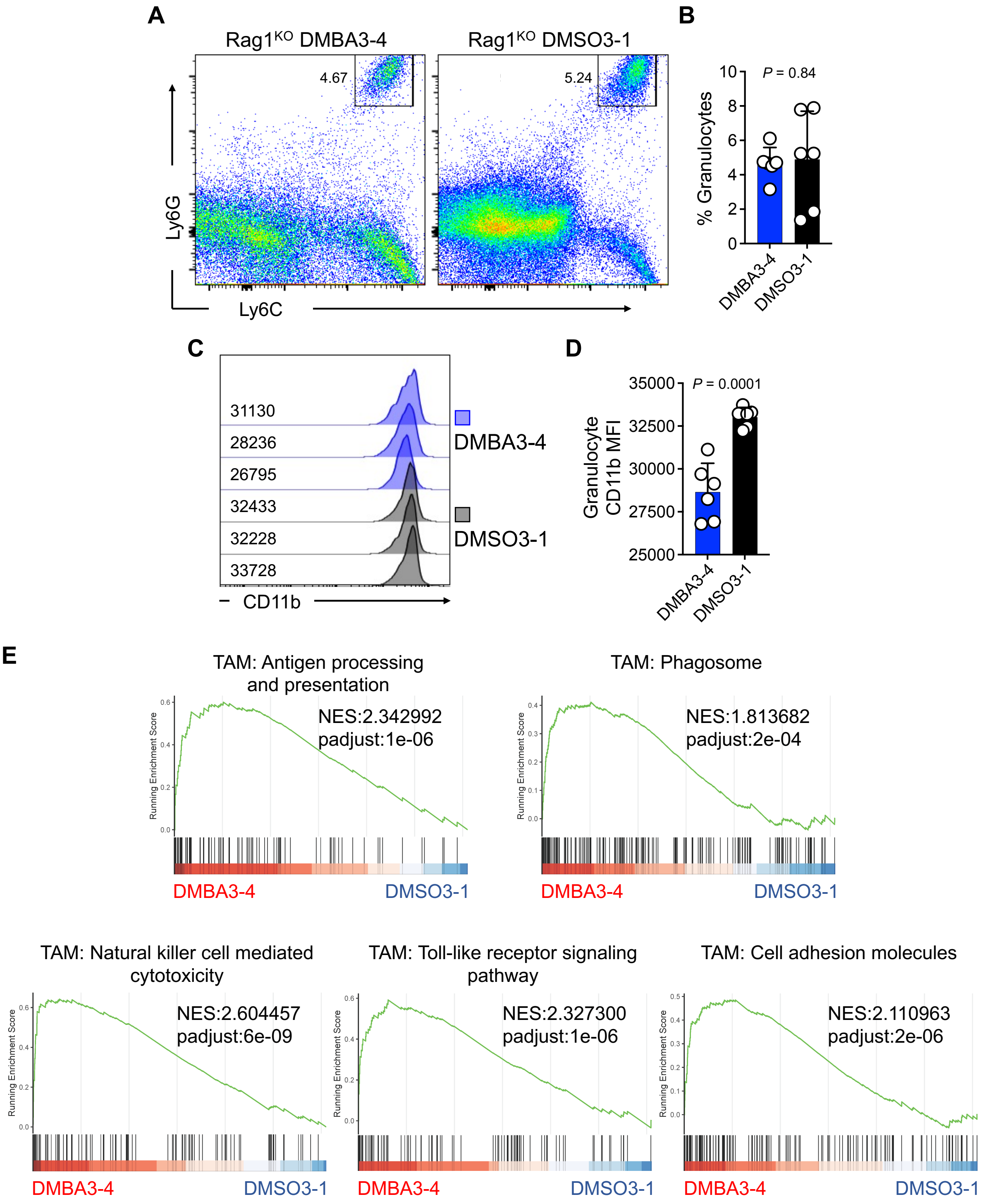

Supplemental Figure 5. Granulocyte and TAM characterization in TME of carcinogen-exposed cancer cells.

(**A**) Representative flow cytometric analysis of granulocytes in DMBA3-4 and DMSO3-1 tumors from $Rag1^{KO}$ mice. Numbers on the dot plots represent the percent $Ly6G^+$ $Ly6C^+$ granulocytes of total $CD45^+$ $CD11b^+$ leukocytes. (**B**) $Ly6G^+$ $Ly6C^+$ granulocyte frequencies of total $CD45^+$ $CD11b^+$ leukocytes in DMBA3-4 and DMSO3-1 tumors (n=6 per group). (**C**) CD11b expression on $Ly6G^+$ $Ly6C^+$ granulocytes in DMBA3-4 and DMSO3-1 tumors. Numbers on the flow histograms represent CD11b MFI. (**D**) Quantification of CD11b MFI on granulocytes in DMBA3-4 and DMSO3-1 tumors (n=6 per group). (**E**) GSEA analysis of TAMs in DMBA3-4 compared with DMSO3-1 tumors from $Rag1^{KO}$ mice. Unpaired *t*-test (Supplemental Figure 4, B and D), bar graphs show mean + s.d.

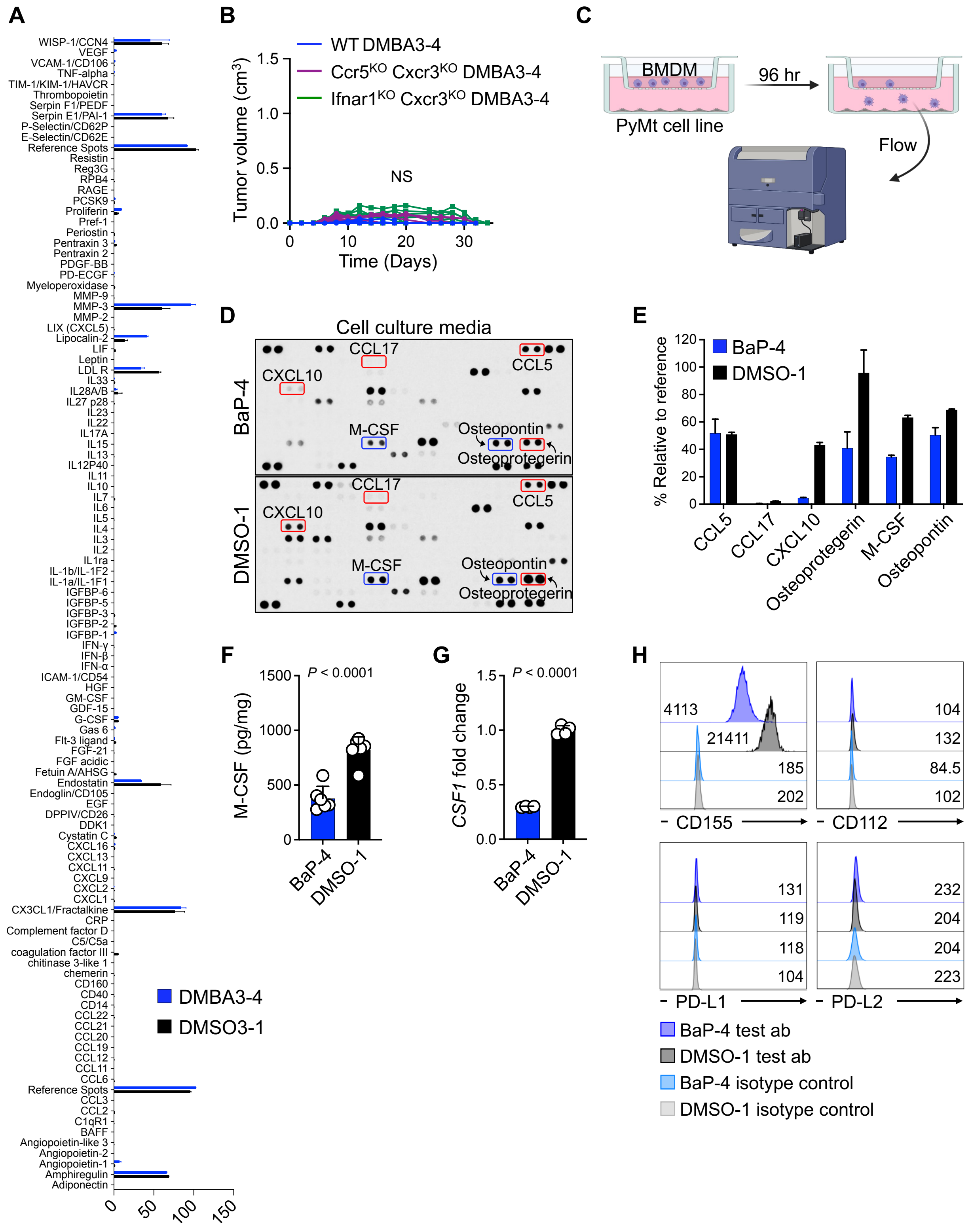

Supplemental Figure 6. Secretome and inhibitory ligand profiling of carcinogen-exposed cancer cells.

(**A**) Relative protein levels from the cytokine array on supernatant from DMBA3-4 and DMSO3-1 cells. (**B**) DMBA3-4 tumor growth in WT (n=6), Ccr5^{KO} Cxcr3^{KO} (n=8) and Ifnar1^{KO} Cxcr3^{KO} mice (n=8). (**C**) Schematic diagram of BMDM migration assay protocol. (**D**) Cytokine array on supernatant from BaP-4 and DMSO-1 cells. Red and blue boxes highlight the upregulated and downregulated proteins from secretome analysis of DMBA3-4 compared with DMSO3-1 cells in Figure 5B, respectively. (**E**) Relative levels of the select proteins from the BaP-4/DMSO-1 cytokine array. (**F**) M-CSF protein levels in BaP-4 compared with DMSO-1 cell lysates (n=6 per group). (**G**) *Csf1* mRNA expression levels in BaP-4 compared with DMSO-1 cells (n=4 per group). (**H**) CD155, CD112, PD-L1 and PD-L2 expression on BaP-4 and DMSO-1 cells. Numbers on the flow histograms represent the ligands' MFI. Two-way ANOVA (NS: not significant, Supplemental Figure 5B) and unpaired *t*-test (Supplemental Figure 5, F and G), bar graphs show mean + s.d.

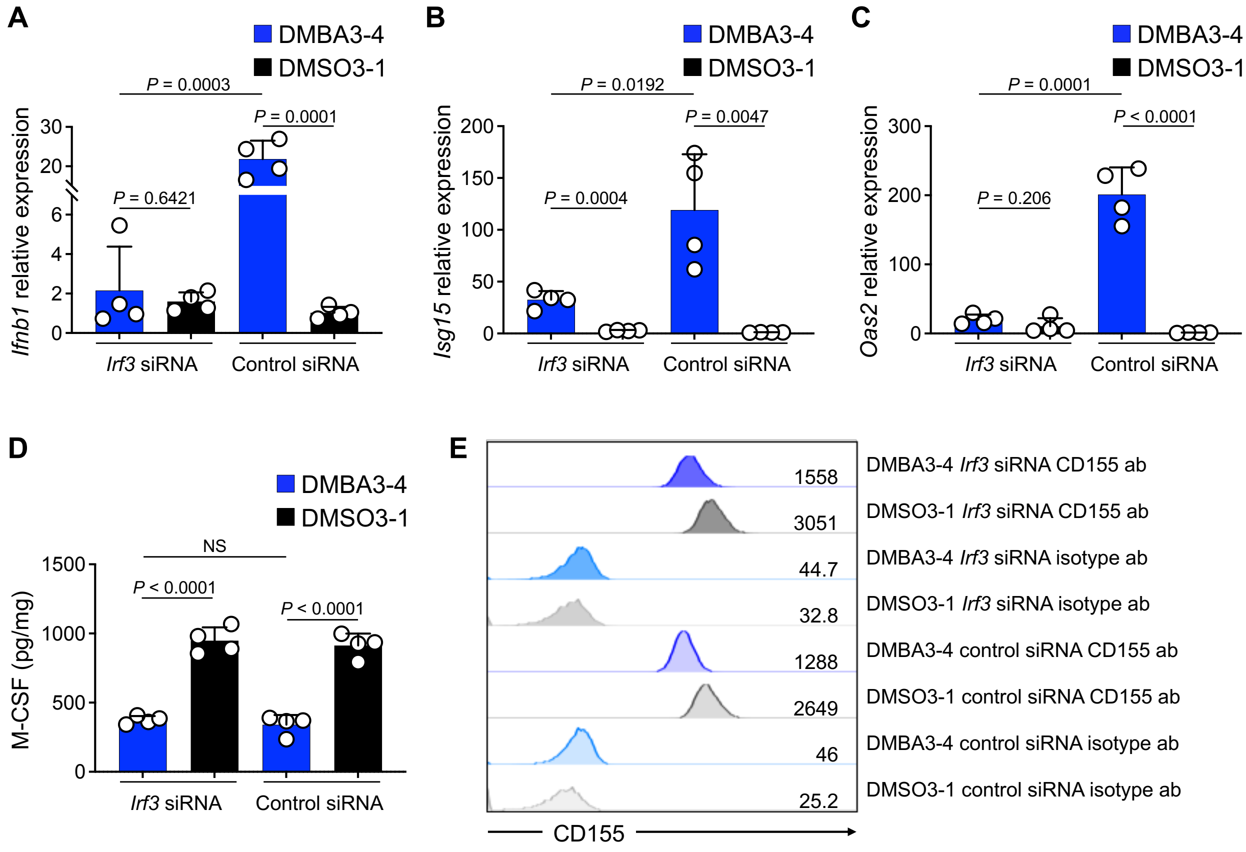

Supplemental Figure 7. Type-I interferon signaling influence on M-CSF and CD155 expression in DMBA3-4 tumor cells.

(**A-C**) The expression of (A) *Ifnb1*, (B) *Isg15,* and (C) *Oas2* in DMBA3-4 and DMSO3-1 cells after transfection with IRF3 versus control siRNA for 24 hours (n=4 per group). (**D**) M-CSF protein levels in DMBA3-4 and DMSO3-1 cell lysates after transfection with IRF3 versus control siRNA for 24 hours (n=4 per group). (**E**) CD155 expression on DMBA3-4 and DMSO3-1 cells after transfection with IRF3 versus control siRNA for 24 hours. Numbers on the flow histograms represent CD155 MFI. Unpaired *t*-test, bar graphs show mean + s.d.

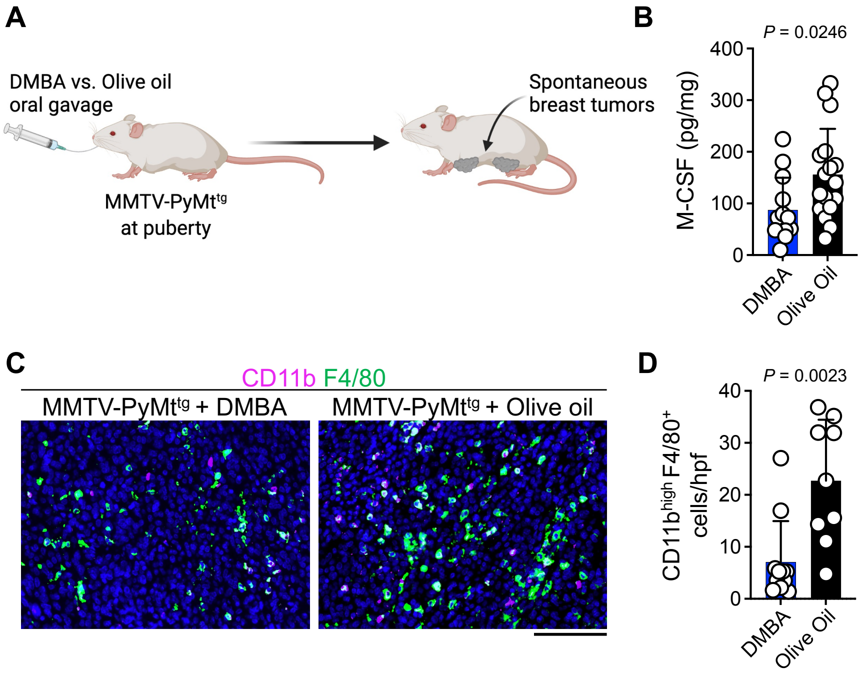

Supplemental Figure 8. The impact of DMBA exposure on the TME of spontaneous breast tumors in MMTV-PyMt^{tg} mice.

(**A**) Schematic illustration of DMBA and olive oil administration to MMTV-PyMt^{tg} mice at puberty and prior to tumor initiation**.** (**B**) M-CSF protein levels in spontaneous breast tumors of DMBA- (n=13) versus olive oil-treated (n=17) MMTV-PyMt^{tg} mice. Each dot represents a tumor sample. (**C**) Representative IF images of CD11b- and F4/80-stained breast tumors from DMBA- and olive oil–treated MMTV-PyMt^{tg} mice (scale bar: 100 μm). (**D**) CD11b^{high} F4/80^{+} TAM counts in breast tumors from DMBA- (n=11) and olive oil–treated (n=9) MMTV-PyMt^{tg} mice. TAMs were quantified in 10 randomly selected hpf images per sample. Each dot represents a tumor sample. Unpaired *t*-test, bar graphs show mean + s.d.

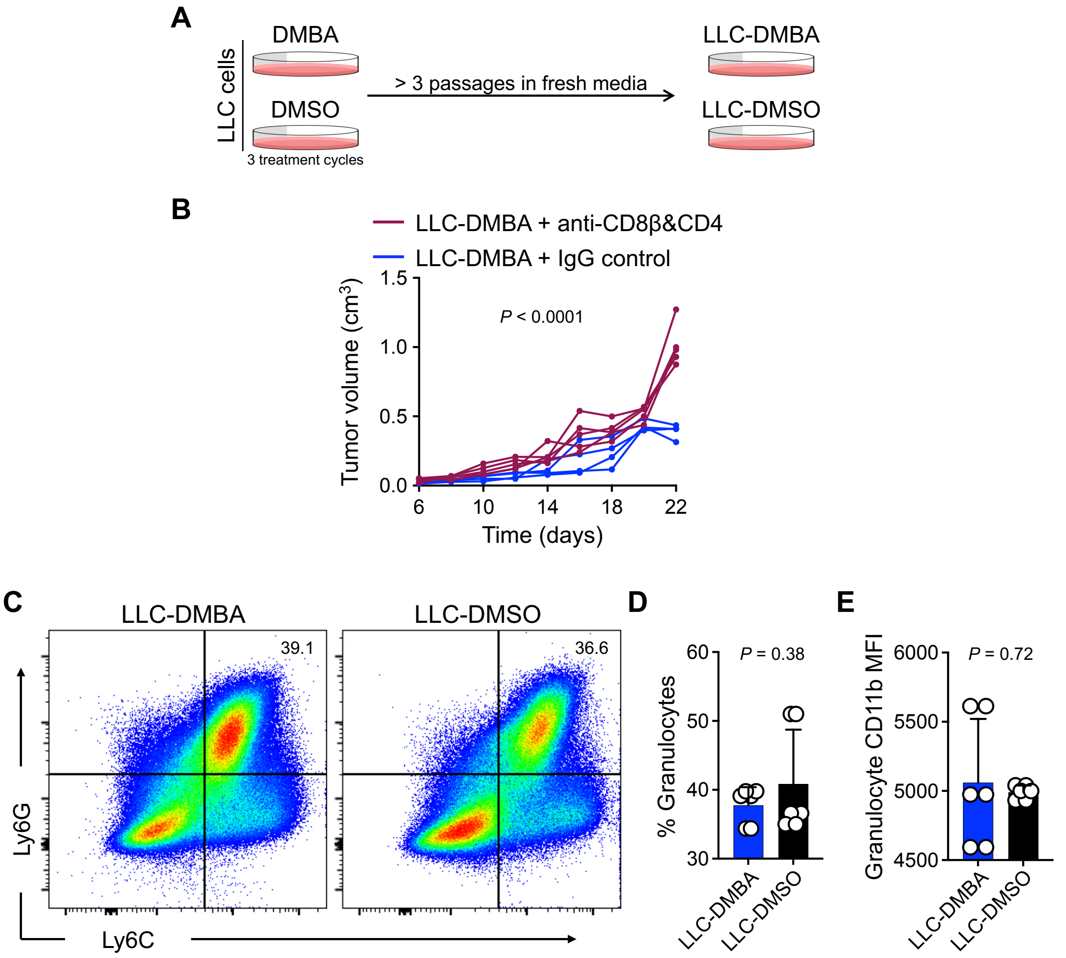

Supplemental Figure 9. Development and characterization of DMBA-exposed LLC cells. (**A**) Schematic diagram of LLC-DMBA and LLC-DMSO cells derived from DMBA and DMSO (vehicle control) treated Lewis lung carcinoma (LLC) cells. Note that LLC-DMBA and LLC-DMSO cells were generated as cell pools (i.e., not single cell clones), which showed a similar proliferation rate *in vitro*. (**B**) LLC-DMBA tumor growth in WT mice treated with anti-CD8β and anti-CD4 combination antibodies (n=5) versus IgG control antibody (n=4). (**C**) Representative flow cytometric analysis of granulocytes in LLC-DMBA and LLC-DMSO tumors from WT mice. Numbers on the dot plots represent the percent Ly6G$^+$ Ly6C$^+$ granulocytes of total CD45$^+$ CD11b$^+$ leukocytes. (**D**) Ly6G$^+$ Ly6C$^+$ granulocyte frequencies of total CD45$^+$ CD11b$^+$ leukocytes in LLC-DMBA and LLC-DMSO tumors (n=6 per group). (**E**) Quantification of CD11b MFI on granulocytes

in LLC-DMBA and LLC-DMSO tumors (n=6 per group). Two-way ANOVA (Supplemental Figure 6B) and unpaired *t*-test (Supplemental Figure 6, D and E), bar graphs show mean + s.d.

Supplemental Table 1. Summary of somatic SNV and InDel mutations in DMBA3-4 compared with DMSO3-1 cells.

Mutations	DMBA3-4 vs. DMSO3-1
CDS	41
synonymous_SNV	7
**missense_SNV**	**0**
frameshift_deletion	1
frameshift_insertion	1
Nonframeshift_deletion	1
Nonframeshift_insertion	0
stopgain	0
stoploss	0
unknown	0
intronic	74
UTR3	12
UTR5	4
splicing	0
ncRNA_exonic	23
ncRNA_intronic	43
ncRNA_UTR3	0
ncRNA_UTR5	0
ncRNA_splicing	0
upstream	3
downstream	5
intergenic	29
**SNV**	**224**
**InDel**	**13**
**Total**	**237**

Supplemental Table 2. Summary of somatic SNV and InDel mutations in BaP-4 compared with DMSO-1 cells.

Mutations	BaP-4 vs. DMSO-1
CDS	137
synonymous_SNV	27
**nonsynonymous_SNV**	**98**
frameshift_deletion	1
frameshift_insertion	0
nonframeshift_deletion	1
nonframeshift_insertion	0
stopgain	9
stoploss	0
unknown	1
intronic	163
UTR3	9
UTR5	10
splicing	3
ncRNA_exonic	32
ncRNA_intronic	63
ncRNA_UTR3	0
ncRNA_UTR5	0
ncRNA_splicing	0
upstream	15
downstream	12
intergenic	26
**SNV**	**413**
**InDel**	**57**
**Total**	**470**

Supplemental Table 3. Antibodies used in the study.

Flow cytometry Antibodies	Conjugate	Clone	Manufacturer	Cat #	Isotype	RRID
CD45	Brilliant Violet 605	30-F11	BioLegend	103155	Rat IgG2b, κ	AB_2650656
CD11b	BUV395	M1/70	BD biosciences	563553	Rat IgG2b, κ	AB_2738276
F4/80	PE-Cy7	BM8	BioLegend	123114	Rat IgG2a, κ	AB_893478
NK1.1	APC	PK136	BioLegend	108710	Mouse IgG2a, κ	AB_313397
MHCII	Alexa Fluor® 488	M5/114.15.2	BioLegend	107616	Rat IgG2b, κ	AB_493523
Arginase 1	PE		R&D Systems	IC5868P	Polyclonal Sheep IgG	AB_10718118
CD206	PerCP-Cy5.5	C068C2	BioLegend	141716	Rat IgG2a, κ	AB_2561992
CD86	Alexa Fluor® 700	GL-1	BioLegend	105024	Rat IgG2a, κ	AB_493721
Ly6C	Brilliant Violet 785	HK1.4	BioLegend	128041	Rat IgG2c, κ	AB_2565852
Ly6G	Alexa Fluor® 647	1A8	BioLegend	127610	Rat IgG2a, κ	AB_1134159
CD155	APC	TX56	BioLegend	131510	Rat IgG2a, κ	AB_10645507
CD112	APC	829038	R&D Systems	FAB3869A	Rat IgG2A	
PD-L1	Brilliant Violet 421	10F.9G2	BioLegend	124315	Rat IgG2b, κ	AB_10897097
PD-L2	APC	TY25	BioLegend	107210	Rat IgG2a, κ	AB_2566345
**Immunofluorescence Antibodies**	**Conjugate**	**Clone**	**Manufacturer**	**Cat #**	**Isotype**	**RRID**
CD3	Purified	CD3-12	Abcam	Ab11089	Rat IgG1	AB_2889189
CD4	Purified	EPR19514	Abcam	Ab183685	Rabbit IgG	AB_2686917
CD8a	Purified	D4W2Z	Cell Signaling Technologies	98941	Rabbit IgG	AB_2756376
CD11b	Purified	EPR19387	Abcam	Ab184308	Rabbit IgG	AB_2889154
F4/80	Purified	D2S9Rl	Cell Signaling Technologies	70076S	Rabbit IgG	AB_2799771
**Depleting/Blocking Antibodies**	**Conjugate**	**Clone**	**Manufacturer**	**Cat #**	**Isotype**	**RRID**
*InVivo*MAb anti-mouse CD4	Purified	GK1.5	BioXCell	BE0003	Rat IgG2b, κ	AB_1107636
*InVivo*MAb anti-mouse CD8β	Purified	53-5.8	BioXCell	BE0223	Rat IgG1, κ	AB_2687706
*InVivo*MAb anti-mouse NK1.1	Purified	PK136	BioXCell	BE0036	Mouse IgG2a, κ	AB_1107737
*InVivo*MAb anti-mouse CSF1R	Purified	AFS98	BioXCell	BE0213	Rat IgG2a, κ	AB_2687699
*InVivo*MAb anti-mouse TIGIT	Purified	1G9	BioXCell	BE0274	Rat IgG1, κ	AB_10950522

Supplemental Table 4. Primers used for mouse genotyping and quantitative PCR.

Gene	Forward primer	Reverse primer
PCR genotyping		
$Batf3^{WT}$	CCAGACCTTTGAATGTGTGAGG	GTTGTGAGTCGAAACCACGC
$Bat3^{KO}$	CCAGACCTTTGAATGTGTGAGG	AGATGAAAAGGCAGCAAGTGT
$Ifng^{WT}$	AGAAGTAAGTGGAAGGGCCCAGAAG	AGGGAAACTGGGAGAGGAGAAATAT
$Ifng^{KO}$	CCTTCTATCGCCTTCTTGACG	AGGGAAACTGGGAGAGGAGAAATAT
$Klrk1^{WT}$	CATAAAGTCCGCTTTGATGTTA	GCAGATTCCCAAAATTCTTG
$Klrk1^{KO}$	CATAAAGTCCGCTTTGATGTTA	ATGAACTTCAGGGTCAGCTT
$Ifnar^{WT}$	CGAGGCGAAGTGGTTAAAAG	ACGGATCAACCTCATTCCAC
$Ifnar^{KO}$	CGAGGCGAAGTGGTTAAAAG	AATTCGCCAATGACAAGA CG
$Ccr5^{WT}$	CAGGCAACAGAGACTCTTGG	TCATGTTCTCCTGTGGATCG
$Ccr5^{KO}$	CTTGGGTGGAGAGGCTATTC	AGGTGAGATGACAGGAGATC
$Cxcr3^{WT}$	GCCTTCCTGCTGGCTTGTAT	AGCAGTGCATGTACCCCATG
$Cxcr3^{KO}$	CTTGGGTGGAGAGGCTATTC	AGCAGTGCATGTACCCCATG
$Sting^{WT}$	AGAACGGACAGCCAGTAAGTATACAG	CAATGCTCTCATAGCCTTCACTATC
$Sting^{KO}$	AACTTCCTGACTAGGGGAGGAGTAG	CAATGCTCTCATAGCCTTCACTATC
$Ticam^{WT}$	AGATGGTTCAGCTGGGTGTC	GGTTCTCCGAACACTCAGTC
$Ticam^{KO}$	AGATGGTTCAGCTGGGTGTC	GGTTCTCCGAACACTCAGTT
$Myd88^{WT}$	GTTGTGTGTGTCCGACCGT	GTCAGAAACAACCACCACCATGC
$Myd88^{KO}$	CCACCCTTGATGACCCCCTA	GTCAGAAACAACCACCACCATGC
$Ncr1^{iCre}$	GACCATGATGCTGGGTTTGGCCCAGATG	ATGCGGTGGGCTCTATGGCTTCTG
$Cd11c\text{-}DTR^{WT}$	ACAACAGAAATCACCCTGGA	TGGCAGTGTTAAAATGCAGA
$Cd11c\text{-}DTR^{Mut}$	ACAACAGAAATCACCCTGGA	CGAGAGGACCTCAGACTGCT
$Rosa\ iDTR^{WT}$	AAAGTCGCTCTGAGTTGTTAT	GGAGCGGGAGAAATGGATATG
$Rosa\ iDTR^{Mut}$	AAAGTCGCTCTGAGTTGTTAT	GCGAAGAGTTTGTCCTCAACC
qPCR		
Csf1	CGGGCATCATCCTAGTCTTGCTGACTGT	ATAGTGGCAGTATGTGGGGGGCATCCTC
Ifnb1	CAGCTCCAAGAAAGGACGAAC	GGCAGTGTAACTCTTCTGCAT
Isg15	GGTGTCCGTGACTAACTCCAT	TGGAAAGGGTAAGACCGTCCT
Oas2	TTGAAGAGGAATACATGCGGAAG	GGGTCTGCATTACTGGCACTT
Gapdh	AATGTGTCCGTCGTGGATCTGA	GATGCCTGCTTCACCACCTTCT
